# EHRA expert consensus document on the management of arrhythmias in frailty syndrome, endorsed by the Heart Rhythm Society (HRS), Asia Pacific Heart Rhythm Society (APHRS), Latin America Heart Rhythm Society (LAHRS), and Cardiac Arrhythmia Society of Southern Africa (CASSA)

**DOI:** 10.1093/europace/euac123

**Published:** 2023-04-15

**Authors:** Irina Savelieva, Stefano Fumagalli, Rose Anne Kenny, Stefan Anker, Athanase Benetos, Giuseppe Boriani, Jared Bunch, Nikolaos Dagres, Sergio Dubner, Laurent Fauchier, Luigi Ferrucci, Carsten Israel, Hooman Kamel, Deirdre A Lane, Gregory Y H Lip, Niccolò Marchionni, Israel Obel, Ken Okumura, Brian Olshansky, Tatjana Potpara, Martin K Stiles, Juan Tamargo, Andrea Ungar, Jedrzej Kosiuk, Torben Bjerregaard Larsen, Borislav Dinov, Heidi Estner, Rodrigue Garcia, Francisco Manuel Moscoso Costa, Rachel Lampert, Yenn-Jiang Lin, Ashley Chin, Heliodoro Antonio Rodriguez, Timo Strandberg, Tomasz Grodzicki

**Affiliations:** Cardiovascular Clinical Academic Group, Molecular and Clinical Sciences Research Institute, St George's University of London, London, UK; Department of Experimental and Clinical Medicine, Geriatric Intensive Care Unit and Geriatric Arrhythmia Unit, University of Florence and AOU Careggi, Florence, Italy; Mercer’s Institute for Successful Ageing, Department of Medical Gerontology, St James’s Hospital, Dublin, Ireland; Department of Cardiology (CVK), Germany; Berlin-Brandenburg Center for Regenerative Therapies (BCRT), Germany; German Centre for Cardiovascular Research (DZHK) partner site Berlin, Germany; Charité Universitätsmedizin Berlin, Germany; Department of Geriatric Medicine CHRU de Nancy and INSERM U1116, Université de Lorraine, Nancy, France; Cardiology Division, Department of Biomedical, Metabolic and Neural Sciences, University of Modena and Reggio Emilia, Policlinico di Modena, Modena, Italy; (HRS representative): Intermountain Medical Center, Cardiology Department, Salt Lake City, Utah, USA; Stanford University, Department of Internal Medicine, Palo Alto, CA, USA; Heart Center Leipzig, Department of Electrophysiology, Leipzig, Germany; (LAHRS representative): Clinica Suizo Argentina, Cardiology Department, Buenos Aires Capital Federal, Argentina; Centre Hospitalier Universitaire Trousseau et Université François Rabelais, Tours, France; National Institute on Aging, MD, USA; Evangelisches Krankenhaus Bielefeld GmbH, Bielefeld, Germany; Department of Neurology, Weill Cornell Medical College, New York, NY, USA; Liverpool Centre for Cardiovascular Science, University of Liverpool, Liverpool, United Kingdom; Department of Cardiovascular and Metabolic Medicine, Institute of Life Course and Medical Sciences, University of Liverpool, Liverpool, United Kingdom; Liverpool Heart and Chest Hospital, Liverpool, United Kingdom; Aalborg Thrombosis Research Unit, Department of Clinical Medicine, Aalborg University, Aalborg, Denmark; Liverpool Centre for Cardiovascular Science, University of Liverpool, Liverpool, United Kingdom; Department of Cardiovascular and Metabolic Medicine, Institute of Life Course and Medical Sciences, University of Liverpool, Liverpool, United Kingdom; Liverpool Heart and Chest Hospital, Liverpool, United Kingdom; Aalborg Thrombosis Research Unit, Department of Clinical Medicine, Aalborg University, Aalborg, Denmark; Department of Experimental and Clinical Medicine, General Cardiology Division, University of Florence and AOU Careggi, Florence, Italy; (CASSA representative): Milpark Hospital, Cardiology Unit, Johannesburg, South Africa; (APHRS representative): Saiseikai Kumamoto Hospital, Kumamoto, Japan; University of Iowa Hospitals and Clinics, Iowa City Iowa, USA; Covenant Hospital, Waterloo, Iowa, USA; Mercy Hospital Mason City, Iowa, USA; School of Medicine, Belgrade University, Serbia; Cardiology Clinic, Clinical Center of Serbia, Serbia; (APHRS representative): Waikato Clinical School, University of Auckland and Waikato Hospital, Hamilton, New Zealand; Department of Pharmacology, School of Medicine, CIBERCV, Universidad Complutense, Madrid, Spain; Department of Experimental and Clinical Medicine, Geriatric Intensive Care Unit and Geriatric Arrhythmia Unit, University of Florence and AOU Careggi, Florence, Italy; Department of Electrophysiology, Heart Center Leipzig, Leipzig, Germany; Department of Cardiology, Aalborg University Hospital, Aalborg, Denmark; Department of Electrophysiology, Heart Center Leipzig, Leipzig, Germany; Klinikum d. Universität München Campus, München, Germany; Chu De Poitiers, Service De Cardiologie, Poitiers, France; Cardiology Department, Hospital Santa Cruz, Lisbon, Portugal; Yale University School of Medicine, Section of Cardiovascular Medicine, CT, USA; Taipei Veterans General Hospital, Cardiology Division, Taipei, Taiwan; UCT Private Academic Hospital, Cape Town, South Africa; Centro Medico Docente la Trinidad, Cardiology Unit, Caracas, Miranda, Venezuela; Department of Health Sciences/Geriatrics, University of Oulu, Oulu, Finland; Jagiellonian University Medical College, Cracow, Poland

**Keywords:** European Heart Rhythm Association, Position paper, Consensus document, Frailty, Elderly, Frailty syndrome, Frailty assessment, Frailty domains, Pre-frailty state, Elderly, Cachexia, Arrhythmias, Atrial fibrillation, Ventricular tachycardia, Heart failure, Stroke, Cognitive impairment, Antiarrhythmic drugs, Anticoagulants, Ablation, Pacemaker, Implantable cardioverter-defibrillator, Cardiac resynchronization therapy, Cardiac resynchronization therapy-defibrillator

## Abstract

There is an increasing proportion of the general population surviving to old age with significant chronic disease, multi-morbidity, and disability. The prevalence of pre-frail state and frailty syndrome increases exponentially with advancing age and is associated with greater morbidity, disability, hospitalization, institutionalization, mortality, and health care resource use. Frailty represents a global problem, making early identification, evaluation, and treatment to prevent the cascade of events leading from functional decline to disability and death, one of the challenges of geriatric and general medicine. Cardiac arrhythmias are common in advancing age, chronic illness, and frailty and include a broad spectrum of rhythm and conduction abnormalities. However, no systematic studies or recommendations on the management of arrhythmias are available specifically for the elderly and frail population, and the uptake of many effective antiarrhythmic therapies in these patients remains the slowest. This European Heart Rhythm Association (EHRA) consensus document focuses on the biology of frailty, common comorbidities, and methods of assessing frailty, in respect to a specific issue of arrhythmias and conduction disease, provide evidence base advice on the management of arrhythmias in patients with frailty syndrome, and identifies knowledge gaps and directions for future research.

## Introduction

Cardiac arrhythmias become more common and embrace a broad spectrum of rhythm and conduction abnormalities with advancing age and chronic illness. According to the World Health Organization, in 2019, 1 billion of people worldwide were aged 60 years and older, with this number set to double by 2050.^1^ The greatest rise is in persons aged 80 years or older, with expected upsurge by four-fold to 434 million worldwide. Concurrently, there is an increasing proportion of the general population surviving with significant chronic disease and disability.

Ageing is frequently characterized by the coexistence of several comorbid conditions, often reciprocally interacting to produce a greater than additive negative impact on health status. Pre-frail state and frailty, mainly in association with old age and multi-morbidity, is increasingly seen among patients with cardiovascular disease and arrhythmias. Management of patients with frailty syndrome has been deliberated in specific settings such as acute cardiac and critical care,^2^ heart failure (HF)^3^ as well as in general cardiology.^4^ Timely and appropriate identification and assessment of the reduction in physiological reserves and frailty, which can be done using the dedicated methods and definitions (see Section ‘Assessment of frailty and frailty scores’) or employing simplified tools based on the characterization of single domains of frailty or self-report questionnaires is useful in guiding the individual approach to patient management and ensuring safe and effective therapies.

However, no systematic studies or recommendations on the management of arrhythmias in the frail population are available, not in the least because these patients have been excluded from major clinical trials, whereas lack of the awareness about the safety and efficacy of antiarrhythmic therapies accounts for withholding effective pharmacological (e.g. anticoagulation) or non-pharmacological (e.g. ablation) interventions. Surveys have revealed a wide variation among physicians in understanding of what constitutes frailty syndrome and highlighted the lack of guidance on the use of the variety of available therapies. Whereas frailty is not synonymous with ageing and the high heterogeneity in older age populations should be acknowledged, practitioners treating arrhythmias will more and more encounter patients who are frail.

Therefore, this consensus document will explain the biology of frailty, common comorbidities, methods of assessing frailty, issues specific to various types of arrhythmias and provide advice on the management of arrhythmias in elderly and frail patients as well as identify knowledge gaps and directions for future research. The document is targeted at primary and secondary care practitioners involved in treating older pre-frail and frail patients with cardiac arrhythmias, conduction disease, and cardiac implanted electronic devices.

## Review of evidence

This consensus document was prepared by the Task Force with representation from European Heart Rhythm Association (EHRA), Heart Rhythm Society (HRS), Asia Pacific Heart Rhythm Society (APHRS), Latin America Heart Rhythm Society (LAHRS), and Cardiac Arrhythmia Society of Southern Africa (CASSA). The document will be peer-reviewed by official external reviewers representing EHRA, HRS, APHRS, LAHRS, and CASSA.

Members of the Task Force were asked to perform a detailed literature review, weigh the strength of evidence for or against a particular treatment or procedure, and include estimates of expected health outcomes where data exist. Patient-specific modifiers, comorbidities, and issues of patient preference that might influence the choice of particular investigations or therapies were considered, as are frequency of follow-up and cost-effectiveness. In controversial areas, or regarding the issues without evidence other than usual clinical practice, a consensus was achieved by agreement of the expert panel after discussions.

Consensus statements are based upon strength of evidence and consensus as outlined in *Table 1*.

**Table 1:** Categories of consensus statements

Consensus statement	Definition	
‘Should do this’	Scientific evidence that a treatment or procedure is beneficial and effective, or is strongly supported by author’s consensus.	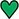
‘May do this’	General agreement and/or scientific evidence favour the usefulness/efficacy of a treatment or procedure	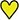
‘Do not do this’	Scientific evidence or general agreement not to use or recommend a treatment or procedure	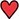

## Relationships with industry and other conflicts of interest

It is EHRA/European Society of Cardiology (ESC) policy to sponsor position papers and guidelines without commercial support, and all members volunteered their time. Thus, all members of the writing group as well as reviewers have disclosed any potential conflict of interest in detail (see Appendix I).

## Definition, epidemiology, and associations of frailty

### Definition and epidemiology

Frailty identifies a syndrome characterized by high biological vulnerability, decreased physiologic reserve, and reduced capacity to resist stressors, due to multiple impairments in inter-related systems, leading to reduced homeostatic reserve.^5^

The prevalence of frailty has been estimated to be 12% (10–15%) in the community, rising to 45% (27–63%) in the non-community cohorts,^6^ with higher rates in women and the highest in individuals aged 85 years and older.^7,8^ Multiple comorbidities are associated with a more than two-fold increase in the prevalence of frailty (up to 63–81%). In analysis including 240 studies from 62 countries representing 1 755 497 participants, the prevalence of frailty in studies using physical frailty measures was 12% compared with 24% for those using a frailty index (FI).^9^ For pre-frailty, this was 46% and 49%, respectively. Physical frailty was more commonly identified in women than men (15% vs. 11%). Combination of multiple comorbidities with frailty phenotype substantially increased long-term mortality risk in septuagenarian men [hazard ratio (HR) 2.93, 95% confidence interval (CI) 2.10–4.07].^10^

Two different conceptual models of frailty have been proposed.^11,12^ According to model proposed by Fried *et al*.,^11^ a cascade of events—from molecular oxidative stress to DNA damage accelerating cellular senescence—leads to endocrine and immune system dysregulation, which results in the development of a frailty phenotype, consisting of reduced muscle strength, body weight, and gait speed, and of increased fatigue or inability to perform demanding activities. An alternative model by Mitnitski *et al*.^12^ describes the cumulative deficit model defines frailty not as a specific syndrome, but rather as an age-related state of additive medical and functional problems. Despite theoretical differences, the two models have much in common and are able to identify older individuals at higher risk of events.^13^

### Pre-frail state

The concept of pre-frail state is less well developed and supported by epidemiological and clinical data, and the definitions are vague. Pre-frail state or intermediate frailty phenotype is identified if one or two out of five criteria based on the Fried model^11^ or as the number of accumulated deficits based on the FI.^14^ It is not uncommon to refer to pre-frailty as a clinically silent phase preceding frailty or a condition that predisposes to frailty.^15^

The exact rates of pre-frailty are difficult to establish. It has been estimated that the prevalence of the pre-frailty state in individuals aged 65 years and older may range between 18.8% and 50.9%. In a recent meta-analysis in the community-dwelling older adults who were robust at baseline 30.9% became pre-frail during a median follow-up of 2.5 years (the incidence rate of 150.6 per 1000 person-years), whereas among non-frail (robust and pre-frail at baseline) individuals, 13.6% progressed to frailty over a medial follow-up of 3 years (the incidence rate of 43.4 per 1000 person-years).^16^ The pre-frailty incidence rates were significantly higher in women than men (173.2 vs. 129.0 per 1000 person-years).

The clinical importance of the pre-frail state concept as a transitional state between robust and frail lies in the possibility of reversal from frailty with effective rehabilitation interventions. Frequent and/or long-term hospitalization associated with sarcopenia and weakness is a major risk factor for transitioning from robust to pre-frail and subsequently developing frailty.

However, the hypothesis that reducing risk factors or enhancing protective factors may prevent or delay age-associated frailty has not been formally tested in appropriately sized randomized controlled trials. There is general consensus that frailty derives from the acceleration of biological ageing and that as our understanding of the biology of ageing progresses, effective prevention and treatment strategies will be developed.

### Knowledge gaps

- Better definition of pre-frail state and tools (derived from the frailty assessment tools or specifically developed) for its evaluation are required.
- Epidemiology of pre-frail state needs to be prospectively studied.
- The driving risk factors associated with gender differences in the incidence and prevalence of pre-frailty and frailty should be explored.
- It is still unknown whether a global care plan that is triggered by the diagnosis of frailty improves objective health outcomes and whether the processes which lead to frailty can be attenuated or reversed is also unknown.
- Physician’s awareness of the importance of evaluation for the pre-frail state and identification of modifiable components with appropriate interventions needs to be improved.

### Frailty vs. clinical complexity

Ageing is associated with progressive loss of biological homeostatic and functional reserve that puts an older individual at high risk of developing adverse health outcomes, including multiple comorbidities, mobility loss, disability, cognitive impairment and eventually becomes so severe to be incompatible with life. While part of this decline of health and functional status is directly attributable to diseases, in some specific individuals the accumulation of damage is so pervasive and multisystemic that it is impossible to recognize a single cause and, therefore, clinicians and scientists define them as frail.

Frailty may have unpredictable trajectories and coexists with other geriatric conditions, such as multiple comorbidities and disability.^17,18^ Ageing is associated with frailty, multiple comorbidities and disability, and these conditions are largely overlapping. Frailty and complexity are sometimes used synonymously, though the term ‘complexity’ should be reserved to indicate the presence of multiple comorbidities with its implicit burden of polypharmacy.^19^ In this respect, complexity may be a component or, better, may contribute to frailty but does not identify with it.

### Major clinical conditions related to frailty

#### Anorexia and malnutrition

Anorexia (loss of appetite or inability to eat), is frequently observed in the older individuals promoting malnutrition and leading through sarcopenia and muscle loss to disability and higher morbidity and mortality. The anorexia of ageing affects approximately 20% of the older population and is higher in hospitalized elderly patients (23–62%) and long-term nursing home residents (up to 85%).^20^ Advanced age often contributes to this cascade through different pathways.^20^ Older patients are less sensitive to the action of ghrelin, the hunger hormone, because of the increased concentration of insulin and leptin, the satiety hormones.^21^ Many conditions that are common in older persons affecting nutritional balance: cancer, HF, chronic obstructive pulmonary disease, chronic kidney disease (CKD), gastrointestinal disorders, mal-absorption syndrome, Parkinson’s disease—all determine anorexia and/or increased energy expenditure. Psychiatric disorders such as depression, cognitive impairment, and dementia also contribute to the reduced appetite. Commonly prescribed medications, swallowing and chewing difficulties, decline of senses, living alone or in a nursing home, and other social or economic problems may negatively influence the nutritional profile. Despite their importance, anorexia and malnutrition are not routinely assessed in everyday clinical practice.

**Table:** Consensus statement

Routine assessment of anorexia and malnutrition and appropriate interventions should be undertaken in all at-risk older persons or other at-risk cohorts.	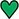

#### Sarcopenia

Sarcopenia, the age-dependent loss of both the muscle mass and muscle strength and function, is a common condition associated with frailty and adverse health outcomes.^22^ After the age of 70 years, the average muscle loss amounts to 15% per each decade. The prevalence of sarcopenia in the community is 1–29% rising to 14–33% in the long-term care. Aging, anorexia, malnutrition, age-related hormonal changes, sedentary lifestyle, and limited mobility mark decreased anabolism, whereas disease state, inflammation, oxidative stress, and mitochondrial dysfunction determine increased catabolism leading to the development of sarcopenia. Sarcopenia should be considered when implementing preventative and therapeutic interventions (e.g. optimized nutrition, elimination of vitamin D deficiency, and physical exercise) aiming at reversing physical frailty at its initial stage and slowing or halting the progressive decline towards disability and dependency.^22^

#### Heart failure

Frailty is particularly common in HF: in a meta-analysis, a 47.4% prevalence of frailty in patients with HF was estimated using multidimensional assessment tools.^23^ The prevalence of frailty in patients with HF is independent of age, suggesting a more complex interaction between the two syndromes and the progressive decline in physiological reserve. The mechanisms linking frailty to HF are multi-factorial, with inflammatory markers, impaired skeletal muscle function due to mitochondrial dysfunction, decreased capillary density, and adipose tissue infiltration potentially involved.^3^ Conversely, ageing, frailty, comorbidities, and immobility due to hospitalization, may all contribute to increase the severity and accelerate the progression of HF leading to increased risk of morbidity and mortality.^24^

**Table:** Consensus statements

Evaluation for frailty should be included into routine clinical management of patients with heart failure.	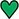
Early intervention targeting modifiable components of frailty is important in improving prognosis and quality of life in heart failure.	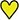

#### Cancer

Frailty is particularly important in the management of cancer patients, who may have mortality rates as high as 80%, as cancer and related therapies may both greatly challenge patient’s physiologic reserve,^25^ when a pre-frail or a clearly frail status can be found in more than 50% of cancer cases.^25,26^ Specific oncologic adverse outcomes possibly related to frailty are chemotherapy side effects,^27^ disease recurrence or progression, and death.^25^ Registry data show that cancer prognosis is associated with frailty-related conditions such as weight loss, reduced gait speed, major depression, and institutionalization in nursing home.^28^ The presence of frailty should promote multidisciplinary decision-making and individually tailored therapeutic approaches aimed at preserving health-related quality of life.

**Table:** Consensus statement

Early evaluation for the signs of frailty and timely recognition of the pre-frail state will enable interventions that may deter progression to frailty and preserve quality of life, particularly in treatable and non-aggressive cancers.	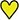

#### Falls

Frail older adults are likely to experience recurrent falls. In the meta-analysis of 102 130 community-dwelling individuals aged 65 years and older, frail patients had a 2.5-fold increased risk of falls, whereas those in the pre-frail state had 1.5-fold increased risk of fall compared with robust individuals.^29^ Frailty-induced falls incur a low quality of life in older adults and increase risk of bone fractures, hospitalization, and death as well as burden on their carers.

Frail patients with suspected arrhythmias should be assessed for falls risk. Modifiable risk factors for falls outwit environmental hazards (loose rugs, steps, etc.) include gait and balance impairment, cognitive impairment, depression, polypharmacy, psychotropic drugs, cardiovascular drugs, visual abnormalities (poor visual acuity and impaired contrast sensitivity), orthostatic hypotension, low blood pressure (BP), arrhythmias (most commonly bradyarrhythmias), urinary incontinence, prior falls, and fear of falling.^30^ All identified risk factors should be modified.

Falls are categorized as accidental, i.e. a slip or trip or non-accidental. The latter are more likely to be attributable to cardiovascular abnormalities, particularly hypotensive disorders or arrhythmias. In practice, the distinction is less clear unless a fall has been witnessed (not the case in up to 50% of events) and/or the older person has a clear recall of events.^31^

**Figure 1:**
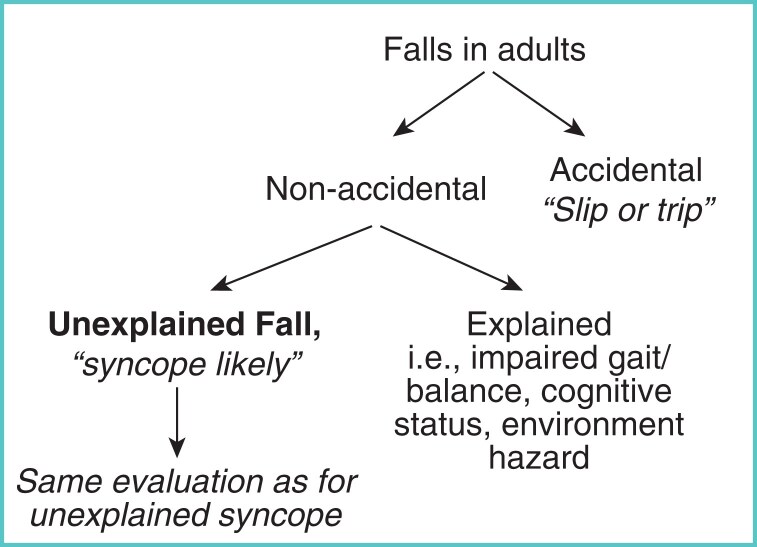
Flow diagram for the identification of unexplained falls. Reproduced from Brignole M, et al. 2018 ESC Guidelines for the diagnosis and management of syncope. European Heart Journal, 2018;39(21):1883–1948, by permission of Oxford University Press and the European Society of Cardiology (ESC).^32^

In older fallers, a detailed cognitive assessment will assist in determining whether the patient’s recall is unreliable, i.e. recall of prodrome, circumstances of fall, loss of consciousness, palpitations, chest pain, post-event characteristics. Such falls should be investigated as per syncope guidelines (*Figure 1*).^32^

In older frail individuals, orthostatic intolerance manifesting as orthostatic hypotension due to pharmacotherapy with antihypertensive and antianginal agents, and sedatives and deconditioning is a common cause of falls. Other causes are primary, secondary autonomic failure, hypovolemia, and anaemia.^33^ Sarcopenia and consequent deconditioning are contributory causes in frail persons.

In one-quarter to one-third of patients with falls, particularly when long-term continuous rhythm monitoring is employed, falls are directly linked with arrhythmias—most commonly bradyarrhythmia, asystole, and tachy-brady forms of atrial fibrillation (AF).^34–36^ Antiarrhythmic medications may potentiate orthostatic intolerance, increase risk of bradycardia, contribute to other falls risk factors, such as impairment of attention/concentration, sleep impairment, electrolyte disturbance, or visual impairment.

**Table:** Consensus statements

Assessment for risk factors for falls is beneficial in all frail patients.	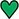
Non-accidental falls which are unexplained should be investigated as per syncope in accordance with the 2018 ESC Guidelines for the diagnosis and management of syncope.	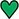
In patients with orthostatic intolerance, precise details of the pharmacotherapy, including non-cardiac medication should be collected.	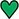
In individuals with low blood pressure and/or orthostatic hypotension/intolerance, medications which lower blood pressure should be used with care and physicians could evaluate possible benefits deriving from medication withdrawal.	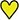
Falls risk should be monitored in patients prescribed antiarrhythmic medications.	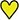
Antiarrhythmic drugs which cause minimal effect on blood pressure are preferred.	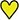

#### Neurological conditions including cognitive impairment and dementia

Frailty and falls are more common in age-related neurological disorders such as stroke, Parkinson’s disease, dementia, or epilepsy.^37^ The proportion of ‘fallers’ is higher even in subgroups with least fall-associated neurological diseases such as tinnitus and headache. Medical treatment, including that for dementia, may also increase the risk of falls through different mechanisms. Psychotropic drugs are related to fall injuries, hospitalization, and death.^38^

Dementia and cognitive impairment play a significant role in this context and are associated with an increased risk of falls. Since prevention of future falls is a major objective of treatment, there is particular interest in the effect of cognitive training on the future risk of falls. As recently shown in meta-analyses of randomized trials, combined exercise and cognitive training improved balance in mild cognitive impairment^39^ and physical exercise had a significant effect in preventing falls in older adults with cognitive impairment.^40^

Another important aspect is the fear of falling in frail patients. Recent studies demonstrated an independent association of frailty with fear of falling, and cognitive behavioural therapies may improve fear of falling.^41^

**Table:** Consensus statements

Patients treated with psychotropic drugs should be monitored for falls.	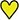
Exercise and cognitive training may improve balance and prevent frailty progression in patients with an early stage of cognitive impairment.	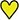

#### Multiple comorbidities and polypharmacy

Cardiovascular Health Study demonstrated a strong association of frailty with a number of chronic diseases including cardiovascular and pulmonary diseases and diabetes: 33% of the frailty subjects had 3–4 chronic diseases, 27% two, and 8% >5 concomitant conditions.^11^ Multiple comorbidities in frailty subjects may not only aggravate frailty phenotypes, but result in the increased risk of polypharmacy which has repeatedly been shown to be a marker of adverse clinical outcome. The French study of a cohort aged >70 years revealed that the mean number of drugs prescribed increased with increasing frailty from 4.6 in non-frail subjects to 6.1 in pre-frail, 7.1 in frail, and 7.5 in dependent individuals.^42^ Importantly, frailty and excessive polypharmacy with >10 drugs were independent risk factors for mortality, and the combination multiplied the mortality risk for 2.6 years by 6.30. HF, renal failure, AF, dementia, and cancer were among the most common comorbidities recognized by physicians as associated with frailty.^43^

#### Electrolyte disorders, renal impairment, metabolic issues

Chronic hyponatremia is the most common electrolyte disorder in the elderly. It is caused mainly by drugs such as diuretics and antidepressants and by syndrome of inappropriate antidiuretic hormone secretion. Though often mild and asymptomatic, it aggravates frailty phenotypes leading to cognitive disorders and gait impairments and contributes to osteoporosis leading to bone frailness, thus predisposing the patients to falls and hip fractures.^44^ CKD is common in ageing. Sarcopenia increases progressively along with renal impairment in CKD, being highest in dialysis patients. Prevalence of frailty has been documented in 7% of the elderly, 14% of CKD patients not requiring dialysis, and 42% of those on haemodialysis.^45^ Frailty in haemodialysis patients is associated with 2.6 times greater risk of mortality and 1.4 times more hospitalization, independent of age, sex, comorbidity, and disability.^46^ Of the metabolic issues, insulin resistance significantly increases with age, and is considered as a major risk factor for many age-related diseases. Insulin resistance plays an important mediating role in the frailty cycle including chronic inflammation, reduced skeletal muscle metabolism, cognitive decline and sarcopenic obesity. Interventions aimed at correcting insulin resistance therefore may have a critical role in preventing or slowing the downward spiral of frail older persons.^47^

## Assessment of frailty and frailty scores

Frailty is considered an age-related syndrome with unknown pathophysiology, operationally defined as loss of functional reserve in multiple physiologic systems, lack of resilience to everyday stressors and elevated risk for a range of adverse health outcomes. Criteria were developed to identify frail older persons in spite of the substantial heterogeneity of this population.

The most popular set of criteria has been developed using data from the Cardiovascular Health Study.^11^ Building on the conceptual framework that frailty derives from a mutually exacerbating cycle of negative energy balance, sarcopenia, and diminished strength and tolerance for exertion, frailty can be defined according to the following criteria: unintentional weight loss, feeling of exhaustion, muscle weakness, slowness while walking, and low levels of activity, ascertained by a combination of self-reported and performance-based measures (*Table 2*). Individuals who have three or more of these criteria should be considered ‘frail’, and those with two of these criteria should be considered ‘pre-frail’. The predictive validity of these criteria toward different outcomes has been overwhelmingly demonstrated in hundreds of manuscripts published in and outside the geriatric literature.

**Table 2:** Diagnostic criteria used for the diagnosis of frailty (Fried criteria)

Measure	Definition
Weight loss	Lost 4.5 kg or more unintentionally over the last year
Exhaustion	Self-report of either ‘felt that everything I did was an effort’ and/or ‘could not get going’ in the last week
Low physical activity	Self-report, equivalent to <90 kCal in women and <128 kCal in men
Slow walking	4 m at usual pace:
	speed <0.76 m/s for height <159 cm in women and <173 cm in men or speed <0.80 m/s for height >159 cm in women and >173 cm in men
Weakness	Grip strength
	Women: <17 kg for BMI <23 kg/m^2^; <17.3 kg for BMI 23.1–26 kg/m^2^; <18 kg for BMI 26.1–29 kg/m^2^; and <21 kg for BMI >29 kg/m^2^
	Men: <29 kg for BMI <24 kg/m^2^; <30 kg for BMI 24.1–26 kg/m^2^; <30 kg for BMI 26.1–28 kg/m^2^; <32 kg for BMI >28 kg/m^2^

Please note diagnostic thresholds for different criteria were modified for different population and different studies. At least 2/5 positive criteria defines pre-frailty and >3/5 criteria defines frailty.

BMI, body mass index.

The second approach is the FI which is calculated as the ratio between the number of deficits detected and the total number of deficits considered, which may be quite variable and include diseases, physical and cognitive impairments, psychosocial risk factors, and geriatric syndromes such as falls, delirium, and urinary incontinence (*Table 3*).^48^ The FI is a strong predictor of adverse health outcomes and allows robust clinical inferences.^49,50^ The FI categorizes older individuals in several classes, from ‘robust’ to ‘severely frail’. Since FI can be generated from almost any set of health-related variables, this tool is highly flexible and can be adapted to the large number of situations and harmonized across research projects and clinical centres.

**Table 3:** Short version of the frailty index

Non-independent functional status History of diabetes mellitus History of either chronic obstructive pulmonary disease or pneumonia History of congestive HF History of myocardial infarction History of percutaneous coronary intervention, cardiac surgery, or angina Hypertension requiring the use of medications Peripheral vascular disease or rest pain Impaired sensorium Transient ischaemic attack or cerebrovascular accident without residual deficit Cerebrovascular accident with deficit

The index is calculated as (total number of variables present)/(total number of variables assessed). A score ≥0.36 indicates frailty.

There are also self-report questionnaires or instruments based on the assessment of single performances (i.e. single domains of frailty).

The most effective clinical use of frailty has been the stratification of patients to verify what medical interventions are beneficial in this special population or whether alternative approaches should be considered. Frail older persons should not be excluded *a priori* from any type of treatment, and decision-making should be treatment-specific and based on scientific evidence. Specifically, where treatment of different types of cardiac arrhythmias have the same beneficial effects in frail and non-frail older persons is unknown.

Overall, the definition of frailty appears to be a powerful tool to establish prognosis, the risk of complication after surgery or aggressive medical interventions as well as forecasting future health care resource utilization. Whether the identification of frailty triggers alternative approaches to care that results in better clinical outcomes remains uncertain.

### Knowledge gaps

- Inconsistency in assessment of frailty by the existing tools, with the physical function and mobility being the only consistent domain, needs to be addressed.
- The need for disease-specific frailty assessment tools should be assessed and methodology for development and validation of such tools should be defined.

## Pathophysiology overview of biological changes associated with frailty

Frailty is a syndrome characterized by decreased reserve and resistance to stressors, causing vulnerability to adverse outcomes such as morbidity, iterative hospitalizations, loss of autonomy and death. Frailty can result from decline across one or more physiologic systems. Schematically, we can distinguish four major domains of frailty:

- Physical, mainly related to loss of muscle mass and function and reduced physical performance.
- Cognitive, due to cognitive decline and/or dementia.
- Psychological, mainly related to depressive manifestations.
- Social, related to isolation and absence of social activities.

### Frailty and age-related electrical and structural changes of the heart and vasculature

Cardiovascular ageing is the result of alterations of the structure and function of:

- Arteries: endothelial dysfunction, intima-media thickness, arterial wall calcifications, alterations in the extracellular matrix.
- Heart: wall hypertrophy and fibrosis, cavities dilation, valve calcifications and degeneration, changes in cardiac muscle cell contractility.

All these alterations concern the totality of the cardiovascular components (large and small arteries, coronary circulation, myocardium, valves, conductive system) and are the pathophysiological background of peripheral vasoconstriction, central artery stiffness, cardiac diastolic and systolic dysfunction. Acute and chronic manifestations of these alterations are very frequent in the elderly, such as systolic hypertension, ischaemic heart disease, arrhythmias, valvular heart disease, stroke, and acute and chronic HF.

### Interaction between ageing cardiovascular system and frailty

Frailty and pre-frailty have been shown to be associated with any type of cardiovascular disease, with an HR of 1.70 (95% CI: 1.18–2.45) and 1.23 (95% CI: 1.07–1.36), respectively, compared with the robust individuals.^51^

The combination of age-related arterial and cardiac alterations and their interactions (cardiac-artery coupling) are responsible for the above-mentioned cardiovascular diseases and also contribute to the development of other age-related degenerative diseases and syndromes such as dementia, sarcopenia, renal failure, leading to frailty and loss of autonomy. Ageing, frailty, and cardiovascular disease are linked by multiple mechanisms and share several biomarkers including inflammation and oxidative stress biomarkers, natriuretic peptides and troponins, and markers of CKD.^52^ Some cardiovascular risk factors such as obesity are important long-term risk factors (already before the appearance of clinical cardiovascular disease) for frailty, particularly in younger individuals.^52^

The extent of cardiovascular ageing which is very variable among older people of the same age, will be a major modulator of functional status, and the degree of functionality and autonomy especially after the age of 80 years.

Older individuals with marked frailty will have a more pronounced evolution of cardiovascular disease. Thus, for the same level of cardiac dysfunction a frail person will have a more pronounced clinical impact than a more robust person. This is very often observed in heart failure patients in whom the presence of frailty and sarcopenia is synergistic with the consequences of heart disease and amplifies clinical signs such as fatigue, dyspnoea, and cachexia. In addition, the presence of frailty will increase the risk of medication-related adverse effects. Clinical studies have shown that the severity of frailty modifies the benefits/risk ratio of several medical and surgical cardiovascular therapies.

**Table:** Consensus statements

It is important to assess the frailty level in older people with cardiovascular disease in order to estimate the risk of functional decline, loss of autonomy, and death.	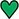
Based on frailty assessment, the risk/benefit balance of therapeutic strategies may be better identified.	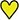
Frailty assessment is necessary in the frame of the holistic management of older patients with cardiovascular diseases especially those with multiple comorbidities and polypharmacy.	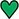
Using age as the main criterion for the provision of health and social care services for older people should be avoided.	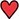

### Knowledge gaps

- Further research is needed to identify biomarkers that may be used for the assessment of interaction between ageing, frailty, and cardiovascular disease and outcomes.

## Clinical pharmacology

Normal ageing produces physioflogical changes which affect the pharmacokinetics (absorption, distribution, metabolism, and excretion) and pharmacodynamics of antiarrhythmic drugs (AADs) (*Table 4*).^53–56^

**Table 4:** Age-associated changes in pharmacokinetics and pharmacodynamics

Pharmacokinetic changes			
	Physiological change	Pharmacokinetic effect	Drugs affected
Absorption	↓ gastric acid production and empting ↓ splanchnic blood flow, motility and absorption surface ↓ first-pass metabolism	antiacids, laxatives can ↓ drug absorption, anticholinergic drugs, propulsive drugs, opioids—can induce OIBD (opioid induced bowel dysfunction)	oral bioavailability of diltiazem, propranolol, verapamil
Distribution	↓ cardiac output and tissue perfusion, peripheral oedema increase Vd, loop diuretics eg furosemide can decrease Vd		
	↓ muscle mass		
	↑ body fat	Vd of highly lipophilic drugs	Vd of highly lipophilic drugs: amiodarone, dronedarone, lidocainne, verapamil
	↓ extracellular and total body water	↓ Vd of hydrophilic drugs	digoxin plasma levels, hydrophilic antiarrhytmic drugs
	↓ plasma albumin, α1-acid glycoprotein	plasma levels of some drugs	free drug levels of amiodarone, diltiazem, dronedarone, propafenone, quinidine, verapamil
Biotransformation	↓ liver mass and hepatic blood flow (20-30%) ↓ CYP450-mediated phase I reactions	exposure of drugs highly biotransformed	amiodarone, diltiazem, flecainide, lidocaine, mexiletine, propafenone, propranolol, quinidine, verapamil
Excretion	↓ renal mass ↓ renal blood flow, GFR and tubular function	exposure and half-life of renally-cleared drugs	exposure to ACEIs, renal eliminated AT1 antagonists, amiodarone, atenolol—beta adrenolytics, digoxin, nadolol, quinidine, sotalol
**Pharmacodynamic changes**			
**Change**		**Consequence**	
Baroreceptor is blunted		Postural hypotension, falls: Class I and IV AADs	
Decreased response to catecholamines		Increased sensitivity to amiodarone, β-blockers and sotalol	
Increased myocardial fibrosis		Decreased conduction velocity (Class I AADs)	
Sinoatrial and atrioventricular node dysfunction		Higher risk of bradycardia and atrioventricular block with Class II and IV AADs and digoxin	
Decreased cardiac reserve		Higher risk of HF with disopyramide and Class IV AADs	
Decreased left ventricular compliance		Decreased cardiac output with Class II AADs	
Increased sensitivity to anticoagulants		Higher risk of bleeding	
Comorbidities and polypharmacy		Increased drug–drug and drug–disease, drug–diet supplements interactions HF decreases hepatic and renal clearance Class II AADs, sotalol and propafenone can exacerbate bronchospasm QT-prolonging drugs increase the risk of torsades—drug interactions	

AADs, antiarrhythmic drugs; ACEIs, angiotensin converting enzyme inhibitors; GFR, glomerular filtration rate; HF, heart failure; Vd, volume of distribution.

### Metabolism

The plasma concentrations of some AADs (propranolol, verapamil) increase due to a decrease in their first-pass metabolism. The increase in body fat increases the volume of distribution (Vd) and the half-life of lipophilic drugs (amiodarone) and the decrease in total body water reduces the Vd and increases the serum concentrations of hydrophilic drugs (digoxin). The decrease in albumin plasma levels increases the free-active fraction of AADs.

Most AADs are biotransformed in the liver by CYP2D6 (flecainide, metoprolol, mexiletine, propafenone, vernakalant), CYP3A4 (amiodarone, diltiazem, dronedarone, quinidine, verapamil) and CYP1A2 and CYP3A4 isoenzymes (lidocaine).^53–57^ Age reduces hepatic blood flow and CYP450 activity increasing the plasma levels and half-lives of AADs metabolized by the liver.^54–58^ Biotransformation of some ADDs (amiodarone, disopyramide, lidocaine, mexiletine, procainamide, propafenone, quinidine, verapamil) produces active metabolites with electrophysiological effects that can be different from those of the parent compound (*N*-acetyl-procainamide is a Class III drug; 5-hydroxypropafenone lacks β-adrenergic blocking effects). Active metabolites explain why the AADs can exert different effects when the drug is administered intravenously or orally.

Age-related reduction in renal blood flow, glomerular filtration rate and tubular secretion decreases the clearance and increases the half-lives of renally-cleared drugs (digoxin, ibutilide, sotalol, and dofetilide).^59^ Other AADs undergo hepatic and renal elimination (dofetilide, procainamide, and disopyramide). Thus, dose adjustment needs to be made for AADs that are directly excreted or whose active metabolites are eliminated by the kidney. Most AADs interact with other widely used drugs. Quinidine, amiodarone, and dronedarone inhibit the P-glycoprotein required for renal excretion of digoxin, thereby increasing its plasma levels. Amiodarone inhibits CYP3A4, CYP2C9, and P-glycoprotein, increasing the plasma levels of drugs widely used in the geriatric population (flecainide, Class II and IV AADs, anticoagulants).^60^

### Adverse effects

Frail patients are more susceptible to some adverse effects of AADs, including bradycardia and atrioventricular (AV) block (Class II and IV AADs or digoxin), intracardiac conduction block (Class I AADs), HF (disopyramide, sotalol, and IV AADs), orthostatic hypotension and falls and urinary retention (Class I A).^53,61,62^ Conversely, older patients present a decreased sensitivity to beta-blockers. The *Beers* criteria recommend to avoid: (i) amiodarone as first-line therapy for AF unless the patient has HF or substantial left ventricular hypertrophy; (ii) disopyramide because of its anticholinergic properties; and (iii) digoxin as first-line therapy for AF or HF and should be prescribed at doses <0.125 mg/day for any indication.^62^

Furthermore, AAD treatment is complicated by coexisting comorbidities (HF, hypertension, coronary artery disease) that affect the pharmacodynamic/pharmacokinetics of AADs and lead to polypharmacy that increases the incidence of adverse effects and drug interactions. Interestingly, several non-cardiovascular QT-prolonging drugs prescribed in the elderly increases the risk of proarrhythmia and should be avoided.

Prescribers should carefully evaluate how age affects the pharmacodynamic/pharmacokinetics of AADs and their possible drug interactions with other drugs widely prescribed in older patients with coexisting comorbidities. Treatment should be started at lower-than-recommended dosages based on hepatic and renal function and gradually titrated until reaching the desired dose, assessing for adverse reactions, mainly proarrhythmic effects.

## Bradyarrhythmias

Bradyarrhythmia incidence is known to increase with age and comorbidity.^63–65^ Therefore, with increasing frailty, more bradyarrhythmias are expected. In a murine model, some animals the same age have reduced heart rate and sinus node function that can be predicted by FI. Furthermore, electrical conduction, action potential morphology and fibrosis are correlated with, and graded by, frailty scores.^63^ Idiopathic degeneration of the sinus node caused by ageing is probably the most common cause of SND.^64^ AV block is more prevalent with advanced ageing. Comparing centenarians with a control group averaging 75 years, first- or second-degree AV block was observed in 25% of the centenarians compared with 7% of the controls.^65^

### Drug-induced bradycardia

Patients with frailty are more often prescribed rate-slowing therapy such as calcium channel blockers, beta-blockers and antiarrhythmics for hypertension, HF, and AF. Frail patients have unpredictable pharmacokinetics due to reduced first-pass metabolism, reduction in muscle mass and worsening renal function leading to adverse effects at standard doses. Even medications within the same class may differ. In the CIBIS Elderly study, patients with HF randomized to bisoprolol or carvedilol had similar incidence of side effects (24–25%). However, bisoprolol conferred greater reduction in heart rate and more dose-limiting bradycardia (bisoprolol 16% vs. carvedilol 11%), whereas carvedilol was associated with shortness of breath (bisoprolol 4% vs. carvedilol 10%).^66^

It is estimated that only 15% of AV block is truly caused by drugs. Although resolution of AV block has been reported in 41% when rate-limiting drugs are discontinued, over half have recurrence of AV block in the absence of therapy.^67^ Patients receiving cholinesterase inhibitors for dementia are more likely to be hospitalized for syncope (HR 1.76) or symptomatic bradycardia (HR 1.69) and to undergo pacemaker implantation (HR 1.49).^68^

### Intraventricular conduction abnormalities

The incidence and prevalence of bundle branch block (BBB) increases with age. For 855 men aged 50 years, prevalence of BBB increased from 1% to 17% over 30 years.^69^ Isolated right BBB is more frequent than left BBB (0.18% vs. 0.1%), increasing with age from 0.4% for 45–54 years to 1.3% for >64 years.^70^ Gender differences are present; BBB has been observed in 11% of men but only 5% of women older than 60 years.^71^ In old and frail patients with syncope and bi-fascicular block, empirical pacemaker implantation can be carried out without the preceding electrophysiological study.^72^

### Pacing: indications, mode selection, programming, follow-up, remote monitoring

Existing guidelines do not recommend pacemaker therapy be altered for the frail patient, other than to consider frailty when considering cardiac resynchronisation therapy (CRT) and to suggest CRT-pacemaker (CRT-P) over CRT-defibrillator (CRT-D), but stress the need for a thorough review of the individual risk-benefit ratio, including the implications of living with a device, and patient preferences.^72,73^ The diagnosis of significant frailty along with other risk factors such as advanced age, limited mobility, and life expectancy-limiting co-morbidities may favour the decision to implant a single-chamber pacemaker.^73^ Untreated SND appears to confer a worse prognosis in the frail. With 17-month follow-up, 57% of patients developed syncope, HF, or AF. Multivariate analysis revealed that age of >65 years was the most important predictor of an event (HR 7.80).^74^ Generally, the risks of pacemaker implantation are similar in younger and older patients, but pneumothorax, lead dislodgement, and erosion due to low body weight are more common in the elderly.^75^ Therefore, given potentially greater benefits of pacing the frail, it is suggested that standard pacing indications are followed, even if taking into consideration the increased complication risk and costs of the procedures.^72,73^

Mode selection depends on the indication for pacing. The UK-PACE trial randomized 2021 patients aged >70 years with high-grade AV block to single- or dual-chamber pacemaker and found no difference in mortality or cardiovascular events.^76^ In very old and/or frail patients with infrequent pauses who have limited functional capacity and/or a short expected survival, the benefit of DDD(R) vs. VVIR pacing is expected to have limited or no clinical impact, and the incremental risk of complications related to the second atrial lead should also be taken into account when choosing the pacing mode.^72^ Conversely, maintaining AV synchrony in patients with SND reduces AF, pacemaker syndrome and HF hospitalizations.^77,78^ Patients with suspected deficits should be formally assessed for frailty using the approved methodology prior to pacemaker implant and selecting the mode of pacing.

For frail patients, attending follow-up may be arduous. With the advent of remote monitoring, in-person follow-ups can be minimized without compromising quality of care. In fact, there is evidence to show remote monitoring can be advantageous.^79^

#### Pacing for undocumented bradycardia

Falls are one of the common features of frailty syndrome. Older patients are likely to have multi-factorial aetiology for falls where it may be difficult to differentiate between mechanical falls and falls due to other causes, e.g. bradyarrhythmic. The differential diagnosis may often be hampered by cognitive impairment and amnesia. In this setting, conventional syncope work-up followed by an implantable loop recorder (ILR) rather than empiric pacemaker implant, is endorsed.^73^

#### Leadless pacing

Leadless pacemakers may prevent some of the complications related to implantation including pre-pectoral haematomas, extrusions, pocket infections, pneumothorax, tamponade and lead dislodgement. Vascular complications because of the large introducer sheaths and pericardial tamponade (around 1%) are significant risks in the older frail patients and need to be considered before implanting a leadless pacemaker.^80^

Over the long-term, the cardiac pacing lead is generally considered the system’s weakest link, with risk of the breakdown of the insulation or wire rupture and infections. The rates of these complications are increased in the presence of concomitant disorders often associated with older age. The absence of a lead decreases the rate of lead-related complications and the system is compatible with magnetic resonance imaging. These features make leadless pacing an attractive option for older and frail patients in need of a pacemaker.^81,82^

Compared to patients with transvenous pacemakers, patients with leadless pacing may experience fewer complications but more dramatic pericardial effusions which highlights the care needed for implantation in older and frail patients. The Micra™ device may have a safety profile similar to that of a transvenous system while providing low and stable pacing thresholds.^81^

The long-term status of these devices is unknown, particularly the risk of endothelialization and fibrosis, which might hamper their extraction. This may need the abandonment of the leadless electrode, which may be a less significant matter for older patients. Currently, up to 25% of patients are paced in VVIR mode, which is particularly suitable in the older and frail patients, likely to undergo few device replacements.

**Table:** Consensus statements

Frailty is common in patients with bradyarrhythmias and usually should not constitute a contraindication to implant of a pacemaker.	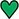
Frailty assessment is required in patients with subtle deficits as it may determine pacing mode selection and follow-up.	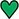

## Ventricular arrhythmias

### Ventricular premature beats and ventricular tachycardia

The incidence of ventricular arrhythmias increases with ageing independently of the presence of underlying heart disease,^83^ and the prevalence of ventricular premature beats (VPB) on Holter ECG in older individuals people has been reported to be as high as 70–80%.^84,85^ Frequent VPBs may occur as a result underlying electrical, structural, ischaemic abnormalities, with different underlying mechanisms such as re-entry involving post-MI scarring, enhanced automaticity in the chronically ischaemic tissue, or triggered activity due to afterdepolarizations associated with acquired QT interval prolongation or caused by digoxin and are associated with increased risk of developing new-onset cardiomyopathy or worsening of the existing disease.^85,86^

Some monomorphic VPBs, most commonly of the right or left ventricular outflow tract origin occur in the absence of structural heart disease, are not associated with an adverse prognosis and usually do not require any specific AAD therapy.^86^

Management of frail patients with frequent VPBs on the background of cardiac pathology, chiefly coronary artery and ischemic heart disease or cardiomyopathy is challenging because of the unfavourable risk/benefit ratio of AAD therapy and only limited data on VPB ablation in this population. Bundle branch re-entry is rare but needs to be identified since it is curable by ablation.

Sudden cardiac death (SCD) in older patients may often be related to malignant ventricular arrhythmias.^87^ The leading cause is myocardial ischaemia, and the prognosis is poor in older patients, with the survival rate <5%.^88^ SCD in old or frail people may also be related to electromechanical dissociation or asystole,^87,88^ which was associated with a nearly 100% mortality rate in most studies. Older survivors may often exhibit cognitive or mood disorders highlighting the influence of age following resuscitated cardiac arrest.^89^

### Pharmacological management of VPBs and ventricular tachycardia

The acute management of ventricular tachycardia (VT) includes intravenous beta-blockers, amiodarone (150–300 mg iv bolus), lidocaine, and mexiletine which may also prevent immediate recurrence of VT and the occurrence of ventricular fibrillation (VF). Amiodarone remains the only AAD that could be used in critically ill patients with frailty.

#### Beta-blockers and non-dihydropyridine calcium antagonists

As mentioned earlier, beta-blockers are often considered first-line treatment in symptomatic patients with a high burden of ventricular ectopy, but their efficacy is modest. In some cases, a non-dihydropyridine calcium antagonist (verapamil) may be used in selected patients and verapamil-sensitive ventricular ectopy. The same therapeutical principles apply to non-sustained ventricular tachycardia (NSVT). Patients with frequent idiopathic runs of NSVT should be evaluated for the primary genetical electrical heart disease.

#### Mexiletine and lidocaine

Mexiletine and lidocaine are effective in suppression of ectopic ventricular automaticity and triggered activity caused by delayed afterdepolarizations, and they also may interfere with the re-entrant mechanism of arrhythmias by converting unidirectional to bidirectional block in partially depolarized myocardium observed during ischaemia. The efficacy of mexiletine as monotherapy was assessed in small studies and during programmed electrical stimulation and ranged between 20–30% in suppressing induced ventricular tachycardia and 75% in reducing the number of NSVT runs.^90^ Mexiletine and lidocaine are similar in both structure and function; unlike lidocaine, mexiletine is well absorbed from the gastrointestinal tract. Mexiletine and lidocaine exert an antiarrhythmic effect without significant inhibition of cardiac function.

Proarrhythmia or other serious toxicity from the drugs is uncommon. Sinus bradycardia and sinus arrest have been documented in patients with pre-existing SND which required monitoring in older frail individuals with a likely impaired sinus and/or AV nodes.

Mexiletine and lidocaine are predominantly metabolized by the liver, and the drug elimination may be delayed in HF and other causes of hepatic insufficiency. For treatment of significant ventricular arrhythmias, mexiletine is given at 200–300 mg tds.; a loading dose of 400 mg can be used followed by 200 mg tds., but the maximum dose should not exceed 1200 mg/day. The elimination half-life of mexiletine is 9–12 h.

#### D, L-Sotalol

Class III (HERG channel-mediated rapid potassium current blockers) D, L-sotalol is generally avoided in frail patients with multiple comorbidities, polypharmacy, and frequent electrolytes disorders, but may be used in selected patients, more commonly for ventricular arrhythmias, if certain provision is followed such as QT interval monitoring and ensuing there is no significant left ventricular hypertrophy. Sotalol also exerts a nonselective competitive beta-1-adrenoceptor antagonism (predominantly confined to the levo-isomer, I-sotalol).^56^ It is effective in suppressing complex forms of ventricular ectopy, displaying superior anti-ectopic activity to beta-blockers in patients with stable coronary artery disease, but is not suitable for patients with HF on the background of hypertensive heart disease and left ventricular hypertrophy and significant left ventricular systolic impairment. Sotalol 160–640 mg/day reduced ventricular ectopy, most notably higher grade ventricular arrhythmias (polymorphic and repetitive premature ventricular complexes, couplets and runs of NSVT); this action was maintained in the presence of mild left ventricular dysfunction and was sustained in the long-term (∼2–6 years).^91^

#### Amiodarone

When beta-blockers alone are ineffective, current evidence suggests that amiodarone is usually beneficial in patients with HFrEF and high VPB burden, also considering its cardiac safety. There are different loading regimens of the drug.

#### Other antiarrhythmic drugs

Based on current evidence, Class IA (disopyramide) and IC (flecainide and propafenone) agents, despite the efficacy and the wide use of the latter in subjects without significant structural heart disease, are not indicated in patients with underlying cardiac conditions and/or HF which are common in frail individuals, because of their negative inotropic effect and risk of ventricular proarrhythmias.^58^ Pure Class III dofetilide is not available worldwide, including many European countries, whereas the use of a multichannel ion blocker dronedarone is limited to AF and is not widely used in Europe.

#### When to intervene?

There is no established clear cut-off point for the initiation of treatment with regard to the number of VPBs. Patients with extremely frequent VPB (>10% of the total beats during 24-h monitoring) are deemed more likely to experience some degree of left ventricular dysfunction or developing arrhythmia-induced cardiomyopathy.^92,93^ A VPB burden of >24% or >20 000 VPBs during the 24-h period has shown a strong association with the development of cardiomyopathy.^94^ However, the threshold varies greatly and may be significantly lower in patients with impaired left ventricular systolic function and HF, with cardiomyopathy and worsening HF being reported in association with VPB frequency as little as 4%.

Other VPB features such as QRS duration as a measure of ventricular dyssynchrony, prematurity index, multiform VPBs, increased VBP density during exercise, repetitive forms such as ventricular couplets and triplets, intrapolated VBPs, VPBs of epicardial origin, and duration of exposure to frequent VPBs may be associated with the development of cardiomyopathy or worsening HF and should prompt further imaging such as cardiac MRI and more intensive follow-up aimed at assessing the frequency of VPBs and left ventricular systolic function (an echocardiogram if MRI did not reveal any underlying condition).

### Drug-induced ventricular arrhythmias

Acquired long QT potentially resulting in polymorphic and torsade de pointes VT is the most significant drug-induced proarrhythmia. The majority of AADs may cause proarrhythmia, particularly in the presence of precipitating factors such as electrolyte abnormalities and drug interaction,^56^ with torsade de pointes considered a significant concern in frail patients where electrolyte disorders and polypharmacy are common. The main cause is iatrogenic QT prolongation (the full list of agents with a torsadogenic potential may be seen at https://crediblemeds.org), but it may also occur when QT prolongation is secondary to sinus bradycardia or AV block. Thus, in the study of 202 hospitalized patients aged above 75 years, 22% of VT cases were ischaemic and 50% were iatrogenic.^84^

#### Treating proarrhythmias

Effective treatment requires the accurate recognition and confirmation of drug-induced proarrhythmia and prompt discontinuation of the implicated agent. Important are also the identification modification of risk factors potentially associated with arrhythmia onset or worsening (e.g. female gender, advanced age, renal or liver dysfunction, underlying structural heart disease, hypokalaemia, hypomagnesaemia, high drug doses/concentrations, rapid intravenous administration, bradycardia, QT prolongation, and pre-existing channelopathies).^56^

The offending drug should be stopped, and intravenous administration of magnesium sulfate irrespective of serum magnesium levels should be administered (e.g. 2000 mg bolus followed by a second bolus and by continuous infusion if the proarrhythmia persists). Bradycardia and pauses that may trigger torsade de pointes should be reversed by either pacing at >70 bpm or isoproterenol infusion. Hypokalaemia should be corrected, aiming at replenishing serum potassium to the high-normal range (i.e. 4.5–5.0 mEq/L).

Beta-blockers may be used in some circumstances. In milder cases, arrhythmias due to digitalis toxicity can be managed by discontinuation of the drug, potassium supplementation and observation. For digitalis-induced life-threatening arrhythmias, several AADs have been proposed in the past (e.g. phenytoin, lidocaine and beta-blockade). More recently, digitalis-specific antibodies have proven effective in reversing digitalis toxicity by rapidly binding to and acutely lowering serum digitalis. Isoproterenol infusion or cardiac pacing are usually effective when symptomatic bradyarrhythmias secondary to conduction abnormalities occur.

### Ablation for ventricular arrhythmias

In patients with high VPB burden and arrhythmia-induced cardiomyopathy worsening HF, ablation may be a preferred treatment option which has been reported to result in a sustained reduction in the VPB burden and associated with a lower risk of hospitalization for HF, cardiac death, or the need for heart transplant.^95^ However, this intervention has not been explored in prospective studies and is limited in patients with frailty syndrome. Some data available from the retrospective cohorts enrolling older patients ≥70 years with structural heart disease (but without systematic formal frailty assessment) undergoing ablation for VT suggest that older individuals were more likely to sustain peri- procedural complications and had higher in-hospital (4.4% vs. 2.3%; *P* = 0.01) and 1-year mortality (15% vs. 11%; *P* = 0.002) compared with their younger counterparts, but a similar incidence of VT recurrence at 1 year (26% vs. 25%) and time to VT recurrence (280 vs. 289 days).^96^ The absence of VT recurrence during follow-up was strongly associated with improved survival in patients ≥70 years. It should be acknowledged that these data concern older, but not necessarily frail individuals and that these data cannot be extrapolated on patients with frailty. On the other hand, these patients may potentially derive a great benefit from the procedure as it may eliminate the long-term risk of pharmacological rhythm control. While it would be challenging to carry out a prospective trial in this setting, collecting data on VT ablation in frail patients should constitute part of national ablation registries or an international registry should be initiated.

### Knowledge gaps

- Due to multi-morbidity and polypharmacy, the usual risk assessment for proarrhythmias may not be sensitive in patients with frailty syndrome; a standardized protocol specifically developed for frail patients may be required.
- There is very limited data on the efficacy and safety of ablation for ventricular arrhythmias in frail patients; an international registry is needed to accumulate these data in a structured fashion.

**Table:** Consensus statements

If patients are symptomatic with VPBs and/or develop evidence of worsening left ventricular systolic function, appropriate therapy should be commenced immediately	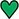
Patients with very frequent VPBs (>20% per 24 hours) at increased risk of developing cardiomyopathy should immediately commence appropriate therapy in order to improve prognosis and prevent the occurrence of cardiomyopathy	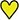
Patients with newly diagnosed frequent VPBs (>500 per 24 h) should be referred for specialist assessment including cardiac imaging (echocardiography, cardiac MRI, exercise stress test, etc.), even asymptomatic, in order to exclude any underlying electrical and/or structural abnormality of the heart	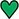
In parallel with specialist electrophysiologic investigation and prior to the initiation of therapy, patients should be carefully assessed for the presence of a frail status; even subtle deficits should be identified and corrected, preferably prior to intervention	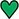
Optimal medical therapy should also aim at underlying heart disease	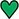
Ablation of frequent VPBs which cause left ventricular systolic dysfunction may be offered to selected patients, after thorough assessment of the risk–benefit ratio of this intervention; many patients with frailty syndrome are unlikely to be candidates for ablation	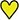

### ICD: indications, selection, and outcome

Implantable cardioverter-defibrillator (ICD) therapy is beneficial in older patients for primary prevention of SCD when life expectancy is >1 year.^97,98^ Randomized studies have reported divergent results on the benefit of ICDs in older patients.^99,100^ In well-selected patients at high risk of arrhythmic death and with few comorbidity factors despite advanced age, ICD intervention may reduce mortality to nearly age-specific life expectancy. It should be acknowledged that these were highly selected populations, and the indications for a primary prevention ICD in the non-ischaemic population is not strong enough to warrant routine ICD implantation in the older and frail patients because of the competing causes for deaths (usually non-cardiac). Thus, in the recent study of octogenarian primary prevention ICD recipients, three quarters had no more than one comorbidity, resulting in similar rates of both appropriate and inappropriate device therapies compared with their younger counterparts.^99^ Among patients who died during 19-month follow-up (35%; of these 38% from non-cardiovascular causes), one-third received at least one appropriate ICD therapy.

In a cohort of 83 792 Medicare patients the NCDR ICD registry, who underwent first primary prevention ICD implantation, approximately 1% had dementia, whereas 10% had frailty defined using a coding algorithm of frailty markers based on 99 ICD-9 codes, selected by a group of geriatricians, and categorized into 10 clusters: malnutrition, dementia, severe vision impairment, decubitus ulcer, incontinence of urine, incontinence of faeces, weight loss, social support needs, difficulty walking, and falls.^100^ Patients with dementia and frailty had substantially higher mortality within the first year after ICD implantation (27% and 22%, respectively) compared with those without these conditions (12%). Several multi-morbidity patterns were associated with high 1-year mortality rates: dementia with frailty (29%), frailty with COPD (25%), and frailty with diabetes (23%).

The predictive usefulness of Charlson Comorbidity Index (CCI) has recently been explored in older (mean age: 78 years) candidates for ICD.^101^ At 5-year follow-up after ICD implantation, survival was 78%, 57%, and 29% in patients with a CCI score of 0–1, 2–3, and ≥4, respectively, compared with 72% in non-frail controls. There was no significant difference in appropriate ICD therapy. The median potential survival gain after an appropriate therapy was >5, 4.7, and 1.4 years, with a CCI score of 0–1, 2–3 and ≥4, respectively. The limitations of CCI include difficulties using in clinical practice, not being developed in older adults, whereas conditions that may have the most bearing on outcomes, such as frailty and dementia, are not incorporated or not appropriately weighted.

Therefore, a multivariable score (rather than chronological age *per se*) and individualized consideration, focusing on comorbidities, projected expectancy, the life-long risk of complications, impact of an ICD on quality of life, including psychological health, and patient preference should help in decision-making for ICD selection and its survival benefit.^102^

In secondary prevention ICD, a meta-analysis suggested that ICD therapy did not show any survival benefit in patients aged >75 years [HR for all-cause death 1.06 (95% CI: 0.69–1.64), HR for arrhythmic death 0.90 (95% CI: 0.42–1.95)].^103^ However, older age does not diminish the likelihood of receiving appropriate therapy.^104^ A careful evaluation of comorbidities that may increase the relative risk of non-arrhythmic mortality is again needed.

In the oldest patients, discussion regarding the effect of implantable devices on the mode of death (ICD preventing SCD but exposing to the risk of prolonged and progressive HF) takes a particular place in decision-making according to patient choice.^105,106^ ICD intervention among the elderly as a group may be less cost-effective, but cost-effectiveness is expected when ICD is implanted in patients expected to live sufficiently, e.g. >5–7 years after implantation.^106^

However, in all these studies much of information comes from carefully selected older individuals with few comorbidities, low-grade frailty, or from mixed populations using heterogeneous definitions for frailty, as this was not the primary endpoint in these studies. The presence of frailty generally was an exclusion criterion from both RCTs and even observational studies. Hence, very limited data exist regarding the risk–benefit of ICD therapy. A review of nine studies, including two RCTs, one prospective cohort, and six retrospective cohort studies, with the number of patients ranging from 77 to 98 437 and follow-up ranging from days to 6 years, has revealed that patients with elements of frailty defined according to varying validated methods, including cumulative deficit models, low weight, and walking speed, had higher all-cause or peri-operative in-hospital mortality.^107^ As mentioned earlier, there is no uniformly established score to assist in identification of a suitable candidate for ICD therapy in frailty, although some physical components, such as a 6-minute walking test, timed chair stands and balance, handgrip strength, as well as questions about weight loss, physical activity, and exhaustion, have been found useful in some reports.^107,108^

Wearable cardioverter-defibrillators may be an alternative to some patients. However, these devices require a high level of compliance, good understanding how it works, and a certain physical strength. Therefore, these devices will have a limited role for prevention of SCD in frail patients.

### ICD programming in frail older patients

Recent ICD studies focused on new programming strategies in patients with little or no exclusion criteria in order to decrease the rate of inappropriate therapies and to deliver less aggressive therapy in case of sustained ventricular arrhythmia.^109–113^ Whether such modern strategies are as safe and efficient in elderly patients is an open question, but such conclusions can be made from these studies. Prolonged detection as well as high VF rate settings decrease the number of inappropriate therapies and the number of shocks with the use of antitachycardia pacing in fast VT. Such programming parameters are proposed in current consensus documents by scientific societies^112,113^ and may be safely applied in elderly or frail patients.

### Subcutaneous ICDs

The subcutaneous ICD (S-ICD) is emerging as a therapy for the prevention of SCD avoiding the complications associated with transvenous leads.^114^ The S-ICD system is essentially promising in terms of reduction of electrode-related complications such as infection or lead failure, which may be more relevant for relatively young and active patients. This may, however, be an option in elderly or frail patients in case of limited vascular access or persistent infection. Low body weight at baseline and risk of progressive body mass and muscle loss, unless revised and corrected, may be the limitation for implanting S-ICD in patients with higher frailty scores.

### Lead failure management

The need for managing CIED infection and abandoned leads increases with advanced age. There are numerous gaps in evidence regarding lead extraction tools, management of abandoned and recalled leads, prevention and treatment of infections, and risk stratification for lead extraction. Such evidence in older and frail patients is difficult to collect because the number of such patients even in high-volume centres, is too low to create statistically solid data. In very old and frail patients with limited life expectancy, leads (and the generator, if therapy is terminated) are usually abandoned.

### Knowledge gaps

- The decision on implantation of an ICD or CRT-D in a patient with frailty should be assisted by a dedicated simple and robust score in order to ensure equal access to therapy for all frail patients; such a score should be developed to be specifically used in people with frailty.

**Table:** Consensus statements

In selected patients at high risk of arrhythmic death and with few comorbidity factors despite advanced age, primary ICD prevention may reduce mortality.	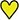
Device programming to deliver optimal ICD therapy aimed at fewer shocks for sustained ventricular arrhythmia may be applied in older frail patients.	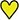

## Heart failure and cardiac resynchronization therapy

### Definition and assessment of frailty in HF

Among all cardiovascular pathologies, HF has the strongest link with frailty, with up to 79% of patients with HF identified as frail.^3,23^ The two conditions share several pathophysiological mechanisms, primarily myocardial and metabolic incompetence, implicated in a so-called ‘dependency cascade’—a process that identifies a sequence of progressive damage involving multiple organ systems that perpetuates itself. ^115,116^ The rates of mortality and hospitalization are the highest in patients with HF who are also identified as frail.

Assessment of frailty is essential in the management of older patients with HF as chronological age does not automatically identifies the health status. Heart Failure Association (HFA) has proposed a definition of frailty in patients with HF as a multidimensional dynamic and partially reversible state, independent of age, that makes the individual with HF more vulnerable to the effect of stressors.^3^ Although several frailty assessment instruments have been used in HF patients,^117^ none of them has been validated in HF. HFA has called for the development of a score that is disease-specific to identify frailty in HF.

### CRT: indications, optimization, follow-up

CRT with (CRT-D) or without defibrillator function is one of the most widely employed non-pharmacological managements of patients with NYHA II–IV class HF, wide QRS complex (primarily LBBB), and left ventricular ejection fraction <35%, with proven efficacy in symptom relief, improvement in exercise capacity and quality of life, hospitalization for HF, and mortality.^72,118^ The prevalence of frailty in patients treated with CRT has not been systematically assessed, but several small studies, using various frailty measurement tools, have reported that it may be as high as 81% in patients undergoing *de novo* implant and 68% in those undergoing the system upgrade.^119^

The benefits of CRT in frail patients are expected to be lower due to multiple comorbidities, including the higher prevalence of AF, although preliminary reports have suggested that the progression in frailty-associated symptoms, such as cognitive impairment, can be deterred by CRT.^120^

Frailty defined by G8 score <14 was associated with a poorer response to CRT and a higher proportion of non-responders.^119,121^ Mortality and hospitalization rates were also significantly higher than in non-frail individuals, with the majority of deaths occurring from HF rather than arrhythmic causes. This underscores the need for systematic screening for frailty in patients at risk who have been offered CRT and the importance of optimal medical therapy and exercise training to reverse or deter frailty-related reduced mobility and nutritional and cognitive impairment.

### Knowledge gaps

- Difficulty assessing frailty in the presence of HF due to the overlap of two conditions, suboptimal performance of frailty assessment tools.
- No reliable tools for the prediction of life expectancy in patients with HF and frailty which is essential for the selection of appropriate therapies (e.g. CRT-D, ICD).
- Lack of data on the outcome of therapies due to the exclusion of elderly frail patients and patients with multiple comorbidities from clinical trials—studies should include the full spectrum of community-dwelling and institutionalized older adults and assess such measures as health status, quality of life, functional capacity, as well as conventional outcomes.
- Further research is needed focusing on the link between mental health (depression) and the clinical course and outcome of HF in association with frailty.
- Need for the improved awareness of frailty and structured management with an emphasis on symptom control, exercise training, and quality of life via rehabilitation programmes.

**Table:** Consensus statements

Targeted rehabilitation programmes focusing on strength, balance, and gait training may be beneficial in deferring the progression of frailty and may partially reverse frailty-associated symptoms and improve quality of life.	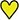
In patients selected or treated with CRT/CRT-D, screening for frailty may be useful in order to assess CRT outcome and the effects of timely applied measures to counteract mobility, nutrition, and cognitive function deficits.	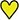

## Device replacement, upgrade/downgrade, and deactivation at end of life

Patients with cardiovascular disease at risk of arrhythmic death and/or heart failure are candidates for ICD and/or CRT, yet, patients with cardiovascular disease are likely to become frail.^23,122^ Clinical effectiveness of ICDs in older populations may be due to “healthy candidate” bias.^123^ Frail patients, being at great risk of death and disability are less desirable candidates for CRT upgrade, ICD implant and/or replacement^106,124^ and many become frail after ICD implant, at time of pulse generator change. A median of 1.2 life-years were gained for those > 80 who came for ICD replacement (2-year mortality 38.1%; 16.7% having life-threatening arrhythmias).^104^ While no prospective trial proves lack of any benefit from ICD replacement or CRT upgrade in a frail population, a substantial percent of frail patients are less likely to respond to CRT (53% vs 73%),^119^ and are at greater risk of heart failure decompensation (55.6% vs. 16.4%),^125^ cardiovascular readmission and death^119,126^ after device implant. Frail patients also have risk of complications including infection, erosion, perforation, and, if necessary, lead extraction with poor outcomes.

Before ICD replacement, at end-of-battery life, frailty (an albeit multifaceted, non-dichotomous condition) should be re-assessed (perhaps in part via activity device monitors^127^) and, whether or not ‘appropriate’ antitachycardia therapies have been given, it is reasonable to forgo ICD replacement and even consider turning off the device.^128^ For those considered for CRT upgrade, similarly, based on the severity of the frailty and underlying comorbidities, it is reasonable to forgo CRT revision.

Because of potentially lower efficacy and higher risk of complications of device therapy, particularly ICD and CRT-D in patients with frailty syndrome, an informative and honest discussion involving legal and ethical issues, including the eventual need for device deactivation should be held with the patient and a caregiver in all individual cases. In frail patients, to improve the dying process, and, with informed, and complete, knowledge of the consequences, it is reasonable for the patient, or legal representative, to request deactivation of any, and all, ICD or CRT and to work with the doctor to ensure this occurs.^128^

However, as generally there is insufficient data, there is also lack of directives regarding advance care planning including device deactivation, palliative, and end-of-life care, whereas lack of training for healthcare providers (e.g. 90% are willing to discuss withdrawal of various life support therapies, but less than 50% would engage in the discussion of ICD deactivation) creates an unmet need in such as sensitive matter.

ICD recipients need education and conversation with their physicians about managing their devices in a systematic fashion. As one survey from octa- and nonagenarian Swedish ICD recipients has revealed, one-third (34%) had discussed their illness trajectory with their physician, a minority (13%) had discussed what turning off shocks would involve with their physician, and just 7% had told their family their wishes about a possible deactivation in the future. About one-fourth of the octo- and nonagenarians had insufficient knowledge regarding the ethical aspects, function of the ICD, and practical consequences of withdrawing the ICD treatment in the end-of-life.^129^ However, it is important that the majority of participants expressed their desire for battery replacement when one was needed, even if they had reached a very advanced age (69%), or were seriously ill with a life-threatening disease (55%).

### Knowledge gaps

- Need for developing a plan for the structured and sensitive discussion involving the physician, the frail patient and his/her family about the possibility of a rapid clinical deterioration, with the onset of a terminal condition and the need for device inactivation and an advance care provision.

**Table:** Consensus statement

An informative and honest discussion involving legal and ethical issues, including the eventual need for device deactivation should be held between the physician, the frail patient, and a caregiver in all individual cases.	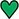

## Supraventricular arrhythmias

Atrial tachycardias are the least frequent form of supraventricular tachycardias in the general population, and there are no specific data in frail patients. There is a higher proportion of macro-re-entrant atrial tachycardias with advance age.^83,130^ Atrial tachycardia is often drug-resistant, whereas ablation may be ineffective due to significant atrial remodelling.

Atrial flutter rates increase significantly with ageing, ranging from 5/100 000 in patients under 50 years of age to 587/100 000 in those aged 80 years and older.^131^ Older frail patients have higher rates of impaired heart rate response and variability characteristics that are further reduced with poor mobility. Although these patients are less likely to be treated with ablation, limited data in selected individuals who were functionally preserved, ablation of typical atrial flutter had a high success rate of 86% and was not associated with excess complications.^132^

Atrioventricular node re-entrant tachycardia can present later in life due to an increase in triggers from ageing and coexistent cardiovascular diseases. Age-related changes in the AV node electrophysiology may lead to prolongation of atrial refractoriness of the slow pathway. No systematic data is available on the efficacy and safety of AV node modification, some limited series reported success rates up to 98%.^133^

## Atrial fibrillation

AF is the most prevalent sustained cardiac arrhythmia in adults, associated in a multifaceted manner with significant morbidity and mortality, hospitalization and impaired quality of life.^134^ Hospitalized AF patients have been shown to have a four-fold greater odds of being classified as frail in comparison to non-AF patients independently of age, sex and comorbidity.^135,136^

The prevalence of frailty in AF patients ranged from 4.4% to 75.4%, while AF prevalence in the frail population ranged from 48.2% to 75.4%.^137^ Among AF patients, frailty was significantly associated with prolonged hospitalization and increased symptom severity, incidence of stroke and all-cause mortality.^136,138^

**Figure 2:**
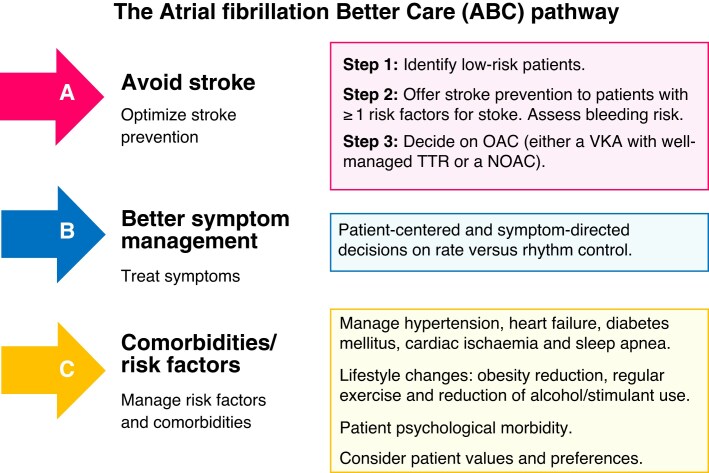
The ABC-integrated AF management pathway. Adapted from Lip GYH.^139^ ABC, Atrial fibrillation Better Care; AF, atrial fibrillation; NOAC, non-vitamin K antagonist oral anticoagulant; OAC, oral anticoagulant; TTR, time in therapeutic range; VKA, vitamin K antagonist.

Owing to a greater prevalence of cognitive impairment, tendency to fall, polypharmacy and cardiovascular or other comorbidity among frail patients, management of AF in such setting may be challenging, since anticipated suboptimal adherence to treatment, drug–drug interactions and increased bleeding risk may influence treatment decisions. The ABC-integrated AF management pathway^139^ (*Figure 2*), that provides a holistic approach to management of AF patients and reminds clinicians of essential decision-making steps in this process, also applies to frail AF patients.

### Rate vs. rhythm control management

There are two primary clinical approaches to managing the arrhythmia.

*Rate control:* slowing the ventricular rate to a level which is physiologically appropriate. Advantages of the rate control approach include ease simplicity avoiding the potential toxicity of AADs or the risks and discomfort associated with electrical cardioversion or invasive left atrial ablation for recurrences of AF.

*Rhythm control:* restoration and long-term maintenance of sinus rhythm; AADs (ion channel blockers) are predominantly used but occasionally autonomic manipulation, for example with beta-blockers may prove valuable.

Rate control remains an essential component of therapy even if the primary strategy is rhythm control (e.g. in the case of a recurrent arrhythmia).

Two targets of rate control have been debated: (i) strict rate control with a target ventricular rate response of <80 bpm at rest and <110 bpm on moderate exercise, usually achieved by the combination of two drugs with an AV blocking effect and repeatedly assessed by different means including an ECG, ambulatory Holter ECG monitoring, exercise stress test, and in some circumstances, implantable rhythm monitoring devices, and (ii) lenient rate control which allows ventricular rates of <110 bpm at rest which usually does not require extensive monitoring.^134^

While lenient rate control with a resting heart rate <110 bpm can be employed as the initial target, it should not lead to the assumption that lenient rate control remains an acceptable, or preferable, option to a more aggressive rate control approach in the long-term. Lenient rate control, often employing a single agent is suitable for older frail individuals with limited morbidity, in whom AF is deemed permanent and is asymptomatic.

Beta-blockers, which are part of the mainstream therapy of HF, represent the first-line choice for acute and long-term rate control, especially in patients with evidence of HF (particularly, HFrEF and HFmrEF), due to their efficacy at high sympathetic drive. Digoxin (62.5–250 µg daily), which has an antiadrenergic effect, increases the AV node refractory period, and inhibits the sodium-potassium adenosine triphosphatase pump—with the resultant improvement in ventricular contractility—can also be used as a first-line rate control drug in permanent AF patients intolerant to beta-blockers, particularly, in older, sedentary, ones. Digoxin is beneficial as a second-line remedy in those with a suboptimal response to beta- or calcium channel blockers. In a small randomized open label study in older patients the majority of whom had HFpEF, digoxin favoured better than bisoprolol in respect of symptom relief and quality of life scores and was associated with a greater adherence to treatment at 6 months.^140^ Digitoxin is a potential alternative to digoxin and is currently being evaluated in a randomized placebo-controlled trial (ClinicalTrials.gov Identifier: NCT03783429). As a last resort amiodarone is advocated, especially in patients with HFrEF.^134^

Of the two prime treatment strategies for AF rhythm control is intuitively more attractive as it offers physiological rate control, normal atrial activation and contraction, the correct sequence of AV activation, normal haemodynamic and AV valve function, and theoretically eliminates one (stasis) or more (endothelial abnormality or increased thrombogenic blood constituents) of Virchow’s triad of elements that encourage thrombosis within the atria and embolization of blood clots to potentially critical parts of the circulation. Elimination of irregular AV conduction, which adds to ventricular dysfunction, is an important component of the beneficial effects of the rhythm control approach. However, the choice of AADs in frail patients with multiple comorbidities, polypharmacy, reduced repolarization reserve, and high risk of proarrhythmia is often limited to amiodarone. The relative cardiac safety of amiodarone should be balanced against significant extra-cardiac side effects. The rate-slowing effect of amiodarone is an additional benefit, particularly in paroxysmal AF.

### Knowledge gaps

- The consequences of the reduction in arrhythmia burden for disability and frailty development or reversal constitute an important knowledge gap in geriatric cardiovascular medicine.

### Stroke prevention

#### General principles

Optimal prevention of stroke or systemic thromboembolism associated with AF involves three crucial steps (*Figure 2*).^139^

Clinician first needs to identify truly low-risk patients (i.e., men with a CHA^2^DS^2^-VASc score of 0 and women with a CHA^2^DS^2^-VASc score of 1 due to female sex), who do not need any antithrombotic therapy, whereas all other AF patients benefit from a stroke prevention strategy, using oral anticoagulant (OAC) drugs [preferably a non-vitamin K antagonist OAC (NOAC), or, alternatively, a well-managed vitamin K antagonist (VKA), with ≥70% time in therapeutic range (TTR)].^134,141^ The preference to NOACs over VKAs largely stems from their better safety, especially concerning haemorrhagic stroke and other intracranial bleeding, and more convenient long-term use in comparison to VKAs.^142^

The concomitant assessment of bleeding risk is needed to control modifiable risk factors and to identify patients with non-modifiable risk factors, who will need frequent clinical follow-up evaluations. The use of the well-validated HAS-BLED bleeding score was superior to the approach concerning only modifiable bleeding risk factors in multiple cohorts,^143–146^ likely due to a more holistic bleeding risk management using the HAS-BLED score. Importantly, neither stroke nor bleeding risk are static, but change over time and need to be re-assessed during clinical follow-up.^134,142^

#### Antithrombotic management in old and frail

A meta-analysis of 6 studies suggested that OAC prescribing patterns in frail AF patients are influenced by a complex interplay of multiple factors including thromboembolic and bleeding risks, frailty status and AF management setting (e.g., the community, hospital or nursing care) reflective of concomitant competing risks, life expectancy, physician’s expertise in AF management and completeness of case assessment.^136^

Factors that may influence OAC under-prescription or discontinuation, such as advanced age, multiple comorbidities, impaired cognitive function, suboptimal adherence and increased bleeding risk,^136,146^ are commonly seen in the general AF population. Regarding advanced age, warfarin was associated with a positive net clinical benefit in AF patients aged ≥75 years in comparison to aspirin [a 2% absolute risk reduction in stroke or systemic embolism, with comparable major bleeding rates (1.4% vs. 1.6%)],^147^ as well as in those ≥90 years old in comparison to no therapy or antiplatelet drugs.^148^

All trials of NOAC treatment in AF included significant populations of older people (defined as ≥75 years) ranging from 31% to 43% in the individual trials, comprising over 27 000 older patients in whom NOACs were studied. In a meta-analysis of pivotal NOAC AF trials, overall efficacy and safety of NOACs were consistent in all age groups,^141^ but there was a significant interaction between age and extracranial major bleeding rates with both dabigatran doses in patients aged ≥80 years (in comparison to warfarin, event rates were similar with the 110 mg dose and significantly higher with the 150 mg dose).^149^ No such interaction was seen with rivaroxaban,^150^ apixaban,^151^ or edoxaban.^152^ Indeed, the higher absolute stroke risk in the elderly resulted in a larger absolute risk reduction with NOACs vs. VKAs and a lower number needed to treat in comparison to younger patients.^152^ Overall, prescribing aspirin instead of OAC in the elderly with AF was actually harmful—the rates of major bleeding with aspirin were similar to those with well-managed VKAs^147^ or NOACs,^153^ whereas aspirin was essentially ineffective in stroke prevention.^154^ Extreme frailty was linked to increased risk of bleeding associated with aspirin.^134^

The use of VKAs was associated with better cognitive function in comparison to aspirin among elderly AF patients, and better cognitive function was also observed among NOAC users compared with warfarin.^155^ In a meta-analysis of five AF studies (one RCT, four observational), OAC use was associated with a significant 21% risk reduction in dementia vs. no antithrombotic therapy, and a TTR of ≥75% was associated with less dementia among warfarin users.^156^ Pivotal NOAC AF trials have not specifically addressed cognitive function, but numerous trials are currently investigating the effects of NOACs on cognitive function in AF patients.

Falls are more common among frail AF patients and are a marker of increased risk of adverse events, but not an independent predictor of OAC-related bleeding, and the net clinical benefit of OAC outweighs the risk of severe bleeding among AF patients who sustained falls. Treatment effects of apixaban and edoxaban were consistent irrespective of the falling risk status, with a larger absolute risk reduction with the respective NOAC vs warfarin, owing to the greater absolute risk of events among patients at increased risk of falls.^157,158^ In a propensity score matched analysis of Medicare data, apixaban was associated with lower rates of adverse events across frailty levels, however residual confounding could not be fully excluded.^159^

Additional considerations when choosing OAC drug (and dose) for a frail AF patient include low body weight (which is a dose-reduction criterion for apixaban and edoxaban), polypharmacy (with increased potential for drug–drug interactions) and comorbidity (i.e. CKD, malignancy, epilepsy, etc.) and are discussed in detail in the EHRA Practical Guide on the use of NOACs in AF patients.^160^ Weight is an important factor affecting the dose selection in a frail patient. Low body weight (usually defined as BMI <18.5 kg/m^2^) may increase exposure to any NOAC and therefore, may increase the risk of bleeding. Body weight ≤ 60 kg requires dose reduction of apixaban [in patients with age ≥ 80 years and/or serum creatinine ≥133 mmol/L (1.5 mg/dL)] as well as for edoxaban, whereas it is in itself not a factor for dose reduction of rivaroxaban or use of lower dose dabigatran. Noteworthy, prescribing a reduced dose of OAC is less effective in preventing AF adverse outcomes.

Notwithstanding all these factors, frailty itself should not preclude the use of OAC (preferably a NOAC) in eligible AF patients. Observational data suggest that among frail AF patients OAC use is associated with lower event rates compared with OAC non-use or aspirin.^136^ In acutely hospitalized frail elderly AF patients, OAC non-use was associated with significantly higher adjusted rate of composite outcome of ischaemic stroke or bleeding compared with OAC use [HR 4.54 (95%CI, 1.83–11.25)],^138^ and in a community-dwelling cohort of older AF patients using OAC, the stroke incident rate was higher than that of major bleeding (1.73 vs. 0.9/100 person-years).^161^ In a retrospective, propensity score-matched analysis of a US administrative dataset of frail AF patients, stroke or systemic embolism rates were significantly lower with rivaroxaban vs. warfarin [1.78 vs. 2.61, HR 0.68 (95%CI 0.49–0.95)] and similar with apixaban (1.68 vs. 2.15) and dabigatran (2.06 vs. 2.20) vs. warfarin, while major bleeding rates were similar with all four OAC drugs and intracranial bleeding was significantly lower with all three NOACs compared with warfarin.^162^

The ELDERCARE-AF trial was a placebo-controlled trial investigating a NOAC (very low-dose edoxaban, 15 mg once daily) in the elderly Japanese patients with AF deemed unsuitable for standard OAC therapy. In this trial, the use of edoxaban was associated with a 4.4%/year absolute risk reduction in stroke at the cost of a non-significant absolute increase in 1.5%/year of major bleeding.^163^

In older patients, the prevalence of cerebral amyloid angiopathy and cerebral microbleeds on MRI is high, and indicates increased risk of intracerebral haemorrhage and is often considered a contraindication for anticoagulation.^134,160^ Whether this risk is lower and the net clinical benefit sufficient to initiate anticoagulation remains unknown, it is advisable to discuss this opportunity on an individual basis.^132^

Importantly, frail AF patients considered for OAC require a detailed assessment of their baseline risk profile and personal values and preferences, as well a frequent clinical follow-up. Patient compliance and adherence, particularly to the twice daily regime, should be assessed at the initiation of therapy and monitored.

#### Left atrial appendage occlusion

In general, the most common justification for left atrial (LAA) closure over systemic OAC is high bleeding risk (e.g. due to falls, liver and kidney dysfunction, polypharmacy and drug interaction) or absolute contraindications to systemic OAC, with frail patients being particularly vulnerable. The decision-making is hindered by insufficient high-quality prospective data comparing LAA occlusion devices with NOACs and the need for antithrombotic therapy after implant and by lack of systematic experience of such an intervention in frail patients, although, according to recent Medicare database analysis, nearly half the patients who underwent LAA closure were considered frail at intermediate or high levels of frailty.^164^ At present, the ESC Guidelines restrict their recommendations for LAA closure therapy to patients with contraindications for long-term OAC, for example, intracranial bleeding without a reversible cause and to patients undergoing cardiac surgery who could have simultaneous surgical LAA occlusion or exclusion.^134^

Although the peri-procedural complications and mortality associated with percutaneous LAA closure is likely to be higher in frailty, this therapy may benefit frail patients in the long-term as analysis of the Medicare database has recently demonstrated. It included 21 787 patients aged 65 years and older who underwent LAA closure, 10 740 (49.3%) of whom were considered frail based on the Hospital Frailty Risk Score (HFRS) >5; 33.5% were regarded as intermediate (HFRS 5–15) and 15.8% as high risk (HFRS >15). HFRS was calculated on the basis of 109 International Classification of Diseases, Tenth Revision, Clinical Modification secondary diagnosis codes from all hospitalizations occurring at least 1 year before the date of admission for the index hospitalization or using secondary diagnosis codes during the index admission.

HFRS >15 was associated with 8.3-fold increased risk of long hospital stay (>10 days) and 1.8 times and nearly 5.7 times higher 30-day readmission and 30-day mortality, respectively, compared with HFRS <5. One-year mortality was increased by 2.8-fold in the high risk group. The one-year mortality rates (8.2%) in frail patients were nearly three- to four-fold higher than reported in the PROTECT-AF (2.5%) and PREVAIL (3.0%) trials.^164^

### Knowledge gaps

- More high-quality data are needed to inform optimal management of stroke risk in frail AF patients.
- A prospective study systematically evaluating the frail status in patients undergoing LAA closure is needed to assess the relationship between frailty and treatment outcomes and benefits.
- Further research is needed for the efficacy and safety of left atrial appendage occluders in high-risk patients (with contraindications for oral anticoagulation) vs. best medical care.

**Table:** Consensus statements

In all AF patients with non-sex-related CHA_2_DS_2_-VASc stroke risk factor(s) OAC therapy is beneficial, irrespective of their frailty status.	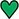
Frail AF patients require a detailed assessment of their baseline stroke and bleeding risk profile and consideration of their personal values and preferences with regard to AF management.	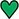
Frailty, cognitive decline and risk of falling is not generally a reason not to anticoagulate patients	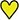
Frail AF patients taking OAC need a frequent, regular clinical follow-up for treatment effects monitoring and stroke and bleeding risk re-assessment.	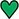
The advantages of NOACs relative to VKAs are likely consistent in frail and non-frail AF patients, but frail AF patients may have a greater absolute benefit from NOACs owing to a higher absolute risk of thromboembolic events in frail AF patients.	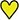
Formal frailty assessment of patients prior to LAA closure may provide important additional information on treatment outcomes and the need for correction of identified deficits and more thorough follow-up.	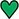
Aspirin should not be used for stroke prevention in frail patients with AF, since it is essentially ineffective and associated with similar risk of bleeding compared with NOACs or VKAs.	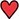

### Ablation: indications and outcome

The frail state, with associated risk factors, can also negatively impact clinical decisions regarding use of more aggressive therapies such as non-pharmacologic interventions, AAD therapy, and anticoagulation.^165,166^ Ablation therapies include AV node ablation and pacing (which has been used predominantly in older patients) and left atrial ablation aiming at rhythm control. Ablation if successful in selected patients with symptomatic AF can provide a durable long-term rhythm benefit without the need for AADs therapies.^167^ Some insight regarding procedural benefit of ablation can be gleaned from examining catheter ablation outcomes in the very aged. Age alone is a significant risk factor for AF recurrence after ablation in older patients.^168^ In patients with successful ablation, the long-term rates of stroke are relatively low across all age groups and associated risk factor profiles. In this regard, patient selection who may benefit from ablation becomes critical.

In observational studies that examined ablation in patients >80 years of age, catheter ablation was safe and effective compared with younger patients. In octogenarian patients, despite more coexisting cardiovascular diseases, 1-year survival free of arrhythmias was 78% vs. 75% in younger patients.^169^ In another study, over a follow-up of 18 ± 6 months, 68% of octogenarians were free of AF compared with 71% of patients that were <80 years of age. In both groups additional procedures increased long-term AF free success rates to 87%.^170^ In both studies severe complication rates were not increased in the older groups. Other studies have addressed slightly younger populations (75 years and older) demonstrating an 86% efficacy rate at 1 year and 52–59% efficacy at 3–5 years.^171–173^ However, older patients are more likely to have non-paroxysmal AF and non-pulmonary vein triggers requiring more extensive left atrial ablation and/or repeated procedures.^169^

There is limited evidence from retrospective reports that some degree of frailty may not be uncommon in patients undergoing AF ablation and that it is associated with higher mortality and adverse outcomes after ablation. Using HFRS calculated from ICD-10 diagnostic codes, 38.6% out of 5070 in-patients treated with catheter ablation were defined as frail with HFRS >5 including 8,3% including 8.3% at high risk (HFRS >15).^172^ Frailty was independently associated with length of stay, post-procedure 30-day mortality, and 30-day readmission rates. The long-term mortality (up to 630 days) was 5.8% in the low-risk group, 23.4% in the intermediate-risk group (HFRS 5-15), and 42.2% in the high-risk group. In propensity score matching analysis, frail patients did not benefit from ablation with respect to HF admissions or stroke as opposed to their non-frail counterparts.^173^

These observational studies represent selected elderly AF patients that were felt to be candidates for the procedure, but in aggregate provide evidence of procedural benefit when ideal candidates are identified. The CABANA trial randomized 2204 AF patients age >65 years, or <65 years with ≥1 risk factor for stroke to antiarrhythmic/rate control drugs vs. catheter ablation. In the intention-to-treat analysis, the primary endpoint of all-cause mortality, disabling stroke, serious bleeding or cardiac arrest (primary endpoint) by intention-to-treat trended towards harm with ablation in those patients >75 years of age [HR 1.46 (95% CI: 0.80–2.67)], however those >75 years of age still were much more likely to remain in sinus rhythm compared with medications [HR 0.48 (95% CI: 0.33–0.68)].^174^

Clearly, some patients, particularly those with multiple other medical conditions, are reluctant to consider a major procedure and have a strong preference for a pharmacological approach. A shared decision-making process is critical in these patients as the disease management is navigated.

### Knowledge gaps

- There is insufficient data on the effect of ablation on mortality, bleeding, and stroke in older patients with multiple comorbidities/pre-frail/frail state.
- Appropriate timing of ablation needs better criteria because older patients are often diagnosed late due to lack of symptoms.
- Type of ablation (pulmonary vein isolation, substrate-based ablation) is not established in the presence of age-related atrial remodelling.

**Table:** Consensus statements

Catheter ablation may be beneficial in selected old and very old patients, particularly if this a patient’s choice and provided that improvement in symptoms and quality of life is expected.	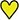
In the majority of frail patients, pharmacological rate control is a preferred option, based on the net clinical benefit, However, an individualized decision process—patient centred—considering the risk/benefit of each therapeutic regimen and patient preference should take place.	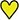

### Silent arrhythmias and screening for AF

AF may often be asymptomatic in up to 40% of cases^175^ or it may present atypical symptoms (in around 25% of cases).^176^ Asymptomatic AF has a higher prevalence in older subjects with permanent AF and is associated with more complex clinical conditions, in terms of comorbidities, that condition a higher thromboembolic risk, resulting in a higher risk of stroke, as well as of cardiovascular and all-cause mortality compared with symptomatic AF.^177^

Detection of asymptomatic (‘silent’) AF, and prescription of oral anticoagulants in patients at risk of thromboembolic events is the target of opportunistic screening.^134^ There is evidence that pharmacy-based, automated AF screening in elderly citizens identified subjects with unknown AF and an excess mortality risk over the next year.^178^ A large variety of devices are currently available for AF screening.^179^ An important aspect of screening is also detection of undertreated known AF, which may be particularly common among frail patients who often do not receive appropriate therapy because of unrecognized AF in 40–50%.^180^

Atrial high-rate episodes (AHREs) detected by CIEDs are usually discovered during routine device follow-up and classified in terms of duration of the single episode or time spent in atrial tachyarrhythmias during a day.^181,182^ These extended diagnostic capabilities have led to new terms, such as ‘AF burden’, defined as the overall time spent in AF during a specified period of time, and ‘subclinical AF’, corresponding to episodes of atrial tachyarrhythmias with duration between 5 min and 24 h, detected by a CIED in patients without clinical history or clinical symptoms of AF.^181^

The prevalence of AHREs or AF burden, among patients implanted with CIEDs varies, depending on underlying heart disease, periods of observation, and above all previous history of clinically overt atrial tachyarrhythmias, including AF.^183^ An analysis of all the data from the literature reveals that that AHREs with a duration >5–6 min are common in patients implanted with CIEDs, with an incidence between 10 and 68%.^184^

The association between CIED-detected atrial tachyarrhythmias of variable durations and stroke or systemic thromboembolism has been evaluated by several studies that overall collected data on >22,000 patients. It has been shown that AHRE burden with a duration ≥5–6 min is significantly associated with increased risk of stroke or systemic thromboembolism, with a hazard ratio ranging between 2 and 9.^180,184^ However, risk of stroke for subclinical AF is around 2.4-fold, i.e. lower than what traditionally reported for clinical AF (4.8-fold).^185^ Therefore, the clinical significance of CIED-detected AHREs with regard to prescription of anticoagulants in terms of risk benefit ratio is currently investigated in prospective trials. The randomized controlled LOOP study has shown that in individuals 70 years of age and older with risk factors for stroke, screening using ILR devices increased the likelihood of AF detection by three-fold accompanied by initiation of OAC therapy, but this strategy was not associated with any significant reduction in rates of stroke or systemic embolism.^186^

Current evidence suggests that a patient-tailored decision-making, particularly in frail patients, is useful when deciding to anticoagulate subjects with AHREs. This approach includes continued patient follow-up, also using remote monitoring of the CIED, targeted to detect the development of clinical AF, to monitor the evolution of AHRE or AF burden and specifically the transition to AHRE lasting more than 24 h, onset or worsening of HF, or any clinical change that might suggest a change in clinical conditions.^187,188^

**Table:** Consensus statements

Asymptomatic (‘silent’) AF detected occasionally and lasting at least 30 s is not a benign condition and requires the same clinical evaluations, with regard to risk stratification for stroke and prescription of antithromboembolic prophylaxis (based on the CHA_2_DS_2_-VASc score), of symptomatic AF	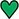
Screen-detected AF, as a result of ECG screening or ECG confirmation as a result of pulse palpation, BP measurement, or apps available in smartphones or watches, is not a benign condition, and after appropriate clinical evaluation and risk stratification for stroke, consideration of antithromboembolic prophylaxis is justified	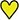
In patients with a cardiac implantable electronic device (CIED) with device-detected atrial tachyarrhythmias (AHREs) a complete cardiological evaluation is indicated, with 12-lead ECG, general assessment of clinical conditions and clinical risk stratification for thromboembolic risk.	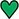
In patients with CIED-detected AHRE, continued patient follow-up—including remote monitoring—is advised to detect the development of clinical AF, to monitor the evolution of AHRE or AF burden and specifically the transition to AHRE lasting more than 24 h, onset or worsening of HF, or any variation that might suggest a change in clinical conditions, as the basis for patient-tailored consideration of oral anticoagulants.	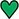

## Stroke as a frailty component and specific characteristics in the very elderly

### Ischaemic vs. haemorrhagic stroke risk

Frail patients face a heightened risk of stroke because they have a substantial burden of vascular risk factors and their clinicians hesitate to treat them with antithrombotic drugs.^189^ Frail patients have worse outcomes than other stroke patients,^190^ and stroke worsens frailty.^191^ Most strokes in AF patients are ischaemic, even in those with an initial haemorrhagic stroke^192^ or a high burden of cerebral microbleeds.^193^

### Implications for acute management and chronic anticoagulation

There is no strong evidence of a net benefit from anticoagulation during the first 14 days after cardioembolic stroke,^194^ although acute anticoagulation appears relatively safe after minor cardioembolic stroke.^195^ A reasonable approach is to initiate anticoagulation within 14 days for most cardioembolic strokes but delay initiation until after 14 days if there is a large infarct, haemorrhagic transformation on neuroimaging, or uncontrolled hypertension.^196^ No good evidence exists to support a different balancing approach in frail patients.

There are few robust data specifically in frail patients regarding chronic anticoagulation for thromboembolism prevention in AF. Advancing age is more strongly associated with thromboembolism than bleeding^197^ and the net clinical benefit of anticoagulation increases with age.^198^ Although physicians hesitate to anticoagulate older patients given fears about falls and intracranial haemorrhage, anticoagulation provides a net benefit even in patients with frequent falls.^199^ There are anticoagulant drugs available that have a similar risk of bleeding as aspirin even in vulnerable patients such as those with prior ischaemic stroke.^200^ These factors support treating frail patients with anticoagulation as otherwise indicated for non-frail patients.

**Table:** Consensus statement

Until further data are available, frail AF patients should receive anticoagulation as otherwise indicated for non-frail patients.	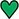

## Orthostatic hypotension and syncope syndrome

### Carotid hypersensitivity syndrome

Carotid sinus syndrome (CSS) is a form of reflex syncope characterized by bradycardia and hypotension. The syndrome is almost exclusively diagnosed in older (male) patients and rare before the age of 40 years. Because a hypersensitive response is present in up to 30% of older patients with syncope, it is recommended that patients above 50 years of age who present with unexplained falls or syncope should have carotid sinus massage (CSM) performed using particular attention in those at risk of neurologic events.^32^ The diagnosis requires reproduction of spontaneous symptoms during asystole and/or a vasodepressor response.^32^ One-third of patients with a hypersensitive response (CSH) to CSM present with unexplained falls; amnesia for loss of consciousness can be reproduced during CSM in such patients.^201^ The finding of a hypersensitive response should not preclude further investigation for other causes of syncope. One-third of CSH patients have other reflex or cardiac abnormalities; conversely, in 80 previously asymptomatic individuals, CSH was present in 28 (35%) and accompanied by symptoms in 10.^202^ The 95th percentile for CSM response in a large random community cohort, mean age 75 years was 7.3 s asystole and a 77 mmHg drop in systolic BP,^202^ suggesting these thresholds for diagnosis and intervention.^203^

### Orthostatic intolerance syndrome

Orthostatic intolerance syndrome is characterized by the abnormal, progressive and sustained drop of systolic and diastolic pressure ≥20 and ≥10 mmHg, respectively, or the decrease in systolic pressure to <90 mmHg.^32^ It can be classified into initial, classical and delayed orthostatic hypotension, according to the time of the onset of the abnormal pressure changes, as described in *Table 5*.

**Table 5:** Syndromes of orthostatic intolerance

Syndrome	Test	Time to abnormal BP response	Pathophysiology	Symptoms	Associated Conditions
Initial OH	Beat-to-beat BP on active standing test	0–15 s	Transient mismatch between cardiac output and total peripheral resistance	Light-headedness, dizziness, visual disturbances few seconds after standing.	Old age, drug-induced (alpha-blockers)
Classical OH	Active standing test; TT	<3 min	Impaired total peripheral resistance and HR increase in autonomic failure; severe volume depletion	Dizziness, light-headedness, weakness, visual and hearing disturbances	Frailty, drug-induced (vasoactive drugs, diuretics), autonomic failure, hypovolaemia
Delayed OH	TT	>3 min	Likely progressive fall in venous return and low cardiac output	Prolonged prodromes: dizziness, light-headedness, visual and hearing disturbances, low back pain, neck or precordial pain	Frailty, incipient autonomic failure, drug-induced (vasoactive drugs and diuretics), comorbidity

BP, blood pressure; ESC, European Society of Cardiology; OH, orthostatic hypotension; TT, tilt testing.

Adapted from 2018 ESC Guidelines for the diagnosis and management of syncope.^32^

### Investigations and management

The diagnostic pathway proposed by the ESC guidelines on syncope,^32^ was used also in older patients, enabling a reduction of unexplained syncope to around 10%.^204^ The initial evaluation relies on clinical history, physical examination, active standing test, and 12-lead ECG. Physicians should search for systemic diseases, physical frailty and locomotor disabilities. Details of cognitive status, social circumstances, injuries, impact of syncope on basal/instrumental activities of daily living, should be recorded. Possible witnesses should be interview, because retrograde amnesia is frequent in older and frail patients. A careful observation of gait and standing balance to assess the risk of falling is mandatory.^32^ Given the high prevalence of CSS in the elderly, the CSM may be performed initially. Even if very rarely (0.24%), the manoeuvre can be complicated by cerebrovascular events. For this reason, it is advised that CSM is undertaken with caution in subjects with a previous TIA or stroke, or when a carotid stenosis >70% is present.^32^ The clinical history has a limited value in the differential diagnosis between cardiac and neurally mediated causes of syncope in older patients,^205^ thus tilt testing (TT) and CSM are essential diagnostic steps.

TT was validated in older subjects^206^ and is well tolerated, even in the oldest old.^207^ TT may detect hypotensive susceptibility, initial and delayed orthostatic hypotension and guide the differential diagnosis between syncope and other clinical conditions causing unexplained falls.

Older patients are more likely to receive an ILR, because of higher rate of structural and electrical heart disease, limited value of the clinical history in determining the syncope aetiology, lack of prodromal symptoms and higher risk of trauma. ILR may be useful in distinguishing between syncope, unexplained falls and epileptic seizures.^36^ In the last few years, technology has allowed to introduce the use of smartwatches in automatic Fall Detection Systems. Benefits in terms of quickness of intervention derive from the widespread acceptance, low cost and networking interfaces of the devices, even if some methodologic aspects are still to be improved.

In older patients with vasodepressor reflex syncope, the recurrence of syncope/pre-syncope may be reduced by discontinuing/reducing vasoactive therapy, without higher adverse events, aimed at achieving an average systolic BP around 140 mmHg, or not <130 mmHg, as recommended in older and frail adults.^208,209^ Medical therapy for orthostatic intolerance includes midodrine, droxidopa, fludrocortisone and piridostigmine, considering supine hypertension.^32,210^ Additionally, isometric physical counter-pressure manoeuvres, and support stockings or abdominal binders to reduce venous pooling, may find an indication to prevent recurrences.^32^

Despite the lack of data on syncope, a comprehensive assessment on cognitive status and physical frailty, may help in the selection of patients for cardiac pacing. According to current ESC Guidelines, dual-chamber cardiac pacing should be considered in subjects with a reflex, asystolic, syncope. A similar level of recommendation can be found also when a cardioinhibitory reflex is dominant in a tilt-induced vasovagal syncope and in a CSS. However, it should be taken into account that the device can significantly reduce the burden of events, but not prevent all of them. Therapy should be also limited to subjects ≥40 years, presenting recurrent episodes with a significant impact on social and active life, when alternative treatment failed or were not feasible.^213^ Moreover, pacing for these specific indications can be found further limitations in older frail individuals.

## Improving patient outcome: specific consideration

### Quality of life

Frailty results in diminished physical, emotional, and social functioning which significantly impacts quality of life.^211,212^ For most frail patients, their desired goals from therapy are improvements in functional status and quality of life, rather than extending life; these patient-centred outcomes should be incorporated into the treatment decision-making process. There is currently a paucity of research specifically examining the impact of frailty per se on quality of life in patients with cardiac arrhythmias.

Frailty significantly impacts treatment choices, with frail patients with cardiac arrhythmias less likely to be aggressively treated compared with their non-frail counterparts.^166^ For example, frail AF patients are less likely to receive oral anticoagulation,^213–215^ primarily due to physicians’ fears of bleeding related to frailty-associated characteristics (risk of falls and cognitive impairment) and are also less likely to receive a rhythm-control strategy.^43,216^ There is also controversy regarding the use of ICDs in frail individuals given the increased risk of non-cardiac death in frail patients that may diminish the benefits of the ICD.^217,218^ However, some types of devices, may be important therapies in frail patients given their impact on outcomes of significance to patients. For example, CRT improves functional capacity, with consequent improvements in physical and cognitive functioning,^120,219^ whilst pacemaker implantation for bradyarrhythmias decreases the risk of falls and reduces frailty,^220^ in addition to preventing onset or worsening of HF, all of which are likely to positively impact overall quality of life.

### Care models

Research suggests that there is little or no evidence for routine comprehensive screening for unmet health needs in the older population.^221,222^ Targeted approaches are better, to identify ‘at-risk’ groups of individuals, with care models focussing on reversing modifiable elements of frailty, particularly addressing underlying medical conditions causing and/or contributing to frailty and optimizing management and reviewing medication, to stabilize the underlying disease processes.

The fundamental issue is that healthcare professionals need to recognize frailty as they are often focussed on specific diseases/comorbidities and these may mask frailty. Education of healthcare professionals is needed to highlight the components of frailty, to acknowledge that frailty is a dynamic process, and demonstrate how to optimize management of this vulnerable patient group. Identification of frailty with an appropriate tool (see Section ‘Assessment of frailty and frailty scores’) should be the first step and if identified, further comprehensive assessment (by a geriatrician) should be undertaken. The concept of frailty requires a more holistic approach rather than a disease-based medical approach and should integrate health and social care and take into consideration the outcomes of importance to patients and their families/carers. As such, this approach requires multidisciplinary interventions and the support and input from a multidisciplinary team.

### Development of a specialist team and aftercare

Respondents of a recent EP Wire,^43^ preferred an ‘Arrhythmia Team’ consisting of an electrophysiologists, clinical cardiologists, geriatricians, internists, palliative care specialists, nurses and family members/carers to manage frail patients. In addition, the specialist team should also include specialist nurses, occupational therapists, physiotherapists/exercise physiologists, general practitioners, pharmacists, and social workers. Composition of the team is largely dependent on the individual needs of the patient; ideally input and care would be provided by all appropriate experts. Ongoing care for frail patients with cardiac arrhythmias is probably best managed by specialist nurse-led aftercare in the community, with appropriate access and referral to a multidisciplinary Arrhythmia team, as required.

Nurses and specialist nurses have an important role in providing a person-centred holistic approach to managing patients with frailty syndrome, monitoring and timely identifying changes in components of frailty syndrome such as assessment of nutrition, pharmacotherapy, adherence to treatment, falls risk, exercise, and mood and cognitive impairment, and prompting interventions to minimize further weight loss, loss of muscle mass and strength, and reduce fall risk factors to help maintain a state of homeostasis.^3^

Patient education and active involvement in decision-making are important for good adherence to OAC and optimal treatment effects.^223^ In addition, various decision supporting tools (built in electronic medical records or mobile applications) have been shown to improve stroke risk management in AF patients.^224,225^

### Digital technology and eHealth

Frail patients who require long-term care with frequent assessment of health state may benefit from digital health technologies (telehealth, mHealth, and wearables such as heart rate and activity tracking and biological sensors).^4^ These technologies allow for for detection and monitoring components of frailty syndrome such as physical activity, gait speed, postural changes and falls, heart rate and fitness, arrhythmias, and assist in early identification of subclinical deterioration in health. Cognitive, visual, or sensory impairments may potentially be assessed through telehealth or mHealth. Telemedicine may enable high-quality and cost-efficient care for an increasing number of patients with frailty. For example, mHealth has become an important component of many AF outpatient clinics in the light of the COVID-19 pandemic and decreased capacity to see patients in the outpatient clinic.^226^ Several validated mHealth solutions are available for remote heart rate and rhythm monitoring as well as for risk factor assessment.^227–229^

## Supplementary Material

euac123_Supplementary_Data
