# Supplementary material for: EHRA expert consensus document on the management of arrhythmias in frailty syndrome, endorsed by the Heart Rhythm Society (HRS), Asia Pacific Heart Rhythm Society (APHRS), Latin America Heart Rhythm Society (LAHRS), and Cardiac Arrhythmia Society of Southern Africa (CASSA)
Source: Europace. 2023 Apr 15;25(4):1249–76. doi: 10.1093/europace/euac123 (PMC10105859; doi:10.1093/europace/euac123)
Supplement: euac123_Supplementary_Data [file euac123_supplementary_data.docx]

	Anker Stefan 2020  Financial Declaration    Direct personal payment from healthcare industry: speaker fees, honoraria, consultancy, advisory board fees, investigator, committee member, etc.For yourself      - BRAHMS GmbH : Biomarker in HF research        - Abbott : COVID-19 SAB        - Janssen / J&J : HF consultancy        - Cordio : HF consultancy        - Servier : HF registry steering committee        - Boehringer-Ingelheim : HF trial executive committee (EMPEROR, EMPERIAL)        - Vifor International : HF trial executive committees (AFFIRM-HF, DIAMOND-HF)        - Cardiac Dimensions : HF trial steering committee        - Bioventrix : HF trial steering committee        - V-Wave : HF trial steering committee        - Bayer AG : HF trial steering committee (DKD)        - Astra Zeneca : SAB in HF (Germany)        - Respicardia : Sleep apnea SAB.    Research funding from healthcare industry under your direct/personal responsibility (to department or institution).For yourself      - Vifor International : CHF therapy development, FAIR-HFpEF & Fair-HF2        - Abbott Vascular : CHF therapy development, RESHAPE-HF2    Other Positions of Influence    Any other interest (financial or otherwise) that should be declared in view of holding an ESC position.For yourself      - Fees for SAB-chair work (<10k EUR / yr) from Actimed Therapeutics, which is a research company (topic: cancer cachexia). I also hold founder's shares of Actimed Therapeutics (<20%).  Benetos Athanase 2020  Financial Declaration    Direct personal payment from healthcare industry: speaker fees, honoraria, consultancy, advisory board fees, investigator, committee member, etc.For yourself      - Novartis : Dementia  Boriani Giuseppe 2020  Financial Declaration    Direct personal payment from healthcare industry: speaker fees, honoraria, consultancy, advisory board fees, investigator, committee member, etc.For yourself      - Bayer : Anticoagulants        - Boston Scientific : Pacemaker, cardioverter defibrillators        - Medtronic : Pacemaker, cardioverter defibrillators Bunch T Jared 2020  Financial Declaration    Research funding from healthcare industry under your direct/personal responsibility (to department or institution).For yourself      - Boehringer-Ingelheim : Atrial Fibrillation        - Boston Scientific : Atrial Fibrillation        - Altathera : Atrial Fibrillation    Other Positions of Influence    Membership or affiliation in political, advocacy or patients organisations working in the field of cardiology.For yourself      - Heart Rhythm Society, American College of Cardiology, American Heart Association Dagres Nikolaos 2020    Nothing to be declared Dubner Sergio Jose 2020  Financial Declaration    Direct personal payment from healthcare industry: speaker fees, honoraria, consultancy, advisory board fees, investigator, committee member, etc.For yourself      - Boehringer-Ingelheim : NOACs    Payment from healthcare industry to your department or institution or any other body for your personal services: speaker fees, honoraria, consultancy, advisory board fees, investigator, committee member, etc.For yourself       - Johnson & Johnson : Fellow grant Fauchier Laurent 2020  Financial Declaration    Direct personal payment from healthcare industry: speaker fees, honoraria, consultancy, advisory board fees, investigator, committee member, etc.For yourself      - Bayer : Healthcare         - Boehringer-Ingelheim : Healthcare        - Medtronic : Healthcare        - Novartis : Healthcare        - BMS/Pfizer : Healthcare        - XO : Healthcare Ferrucci Luigi 2020    Nothing to be declared Fumagalli Stefano 2020  Financial Declaration    Direct personal payment from healthcare industry: speaker fees, honoraria, consultancy, advisory board fees, investigator, committee member, etc.For yourself      - Bruno Farmaceutici : Atrial Fibrillation        - Bayer : Atrial Fibrillation and DOACs        - Boehringer-Ingelheim : Atrial Fibrillation and DOACs        - Pfizer : Atrial Fibrillation and DOACs        - Bristol Myers Squibb : Atrial Fibrillation and DOACs        - Daiichi Sankyo : Atrial Fibrillation, DOACs and Frailty    Travel and meeting support from healthcare industry, independent of the above activities.For yourself      - Daiichi Sankyo : Geriatric Cardiology Israel Carsten W 2020  Financial Declaration    Direct personal payment from healthcare industry: speaker fees, honoraria, consultancy, advisory board fees, investigator, committee member, etc.For yourself      - Berlin Chemie AG : Antiarrhythmic drugs        - BMS-Pfizer : Apixaban        - Astra Zeneca : Brillique        - Impulse Dynamics : Cardiac Contractility Management        - Boehringer-Ingelheim : Dabigatran        - Sanofi Aventis : Dronedarone        - Daiichi Sankyo : Edoxaban        - Servier : Ivabradin        - Zoll Medical : Life Vest        - Boston Scientific : Pacemakers, ICDs        - Biotronik : Pacemakers, ICDs, CRT        - Microport : Pacemakers, ICDs, CRT        - Medtronic : Pacemakers, ICDs, CRT, ILR        - Abbott : Pacemakers, ICDs, CRT, Occluder        - Bayer Healthcare : Rivaroxaban    Payment from healthcare industry to your department or institution or any other body for your personal services: speaker fees, honoraria, consultancy, advisory board fees, investigator, committee member, etc.For yourself       - Astra Zeneca : Brillique        - Zoll Medical : Life Vest        - Medtronic : Pacemakers, ICDs, CRT, ILR    Travel and meeting support from healthcare industry, independent of the above activities.For yourself      - Sanofi Aventis : Antiarrhythmics        - Berlin Chemie AG : Antiarrhythmics        - Boehringer-Ingelheim : oral anticoagulation        - Daiichi Sankyo : oral anticoagulation        - Pfizer : oral anticoagulation        - Bayer Healthcare : oral anticoagulation        - Bristol Myers Squibb : oral anticoagulation        - Abbott : Pacemaker, ICD, CRT        - Boston Scientific : Pacemaker, ICD, CRT        - Medtronic : Pacemaker, ICD, CRT        - Biotronik : Pacemaker, ICD, CRT        - Microport : Pacemaker, ICD, CRT        - Zoll Medical : sudden cardiac death    Research funding from healthcare industry under your direct/personal responsibility (to department or institution).For yourself      - Abbott Vascular : LAA occluders        - Microport : Pacemaker, ICD, CRT    Other Positions of Influence    Membership or affiliation in political, advocacy or patients organisations working in the field of cardiology.For yourself      - Deutsche Herzstiftung    Any other interest (financial or otherwise) that should be declared in view of holding an ESC position.For yourself      - Member of the Board of the World Society of Arrhythmias Editor of "Herzschrittmachertherapie und Elektrophysiologie" Kamel Hooman 2020  Other Positions of Influence    Any other interest (financial or otherwise) that should be declared in view of holding an ESC position.For yourself      - Dr. Kamel serves as a PI for the NIH-funded ARCADIA trial (NINDS U01NS095869) which receives in-kind study drug from the BMS-Pfizer Alliance for Eliquis® and ancillary study support from Roche Diagnostics, serves as Deputy Editor for JAMA Neurology, serves as a steering committee member of Medtronic’s Stroke AF trial (uncompensated), and serves on an endpoint adjudication committee for a trial of empagliflozin for Boehringer-Ingelheim.  Kenny Rose Anne 2020  Financial Declaration    Direct personal payment from healthcare industry: speaker fees, honoraria, consultancy, advisory board fees, investigator, committee member, etc.For yourself      - Medtronic : Syncope Lane Deirdre 2020  Financial Declaration    Direct personal payment from healthcare industry: speaker fees, honoraria, consultancy, advisory board fees, investigator, committee member, etc.For yourself      - Boehringer-Ingelheim : Atrial fibrillation        - Pfizer : Atrial fibrillation        - Bayer Healthcare : Atrial fibrillation    Research funding from healthcare industry under your direct/personal responsibility (to department or institution).For yourself      - Bristol Myers Squibb : Atrial fibrillation and stroke prevention Lip Gregory Yh 2020  Financial Declaration    Payment from healthcare industry to your department or institution or any other body for your personal services: speaker fees, honoraria, consultancy, advisory board fees, investigator, committee member, etc.For yourself       - Daiichi-Sankyo : Anticoagulation        - Bayer/Janssen : Anticoagulation        - Boehringer Ingelheim : Anticoagulation; Registries; Steering Committees        - Pfizer : Anticoagulation; Registries        - BMS : Antithrombotic therapy    Research funding from healthcare industry under your direct/personal responsibility (to department or institution).For yourself      - Boehringer-Ingelheim : AF registries [unrestricted educational grant]    Other Positions of Influence    Any other interest (financial or otherwise) that should be declared in view of holding an ESC position.For yourself      - Shares in private limited company (a legal separate entity in UK), but no salary/dividends/income/personal renumeration received. Marchionni Niccolo' 2020  Financial Declaration    Direct personal payment from healthcare industry: speaker fees, honoraria, consultancy, advisory board fees, investigator, committee member, etc.For yourself      - Bayer : Direct Anticoagulants         - Daiichi Sankyo : Direct Anticoagulants         - Bristol Myers Squibb : Direct Anticoagulants     Research funding from healthcare industry under your direct/personal responsibility (to department or institution).For yourself      - Bristol Myers Squibb : Direct Anticoagulants  Obel Isreal Wp 2020    Nothing to be declared Okumura Ken 2020  Financial Declaration    Direct personal payment from healthcare industry: speaker fees, honoraria, consultancy, advisory board fees, investigator, committee member, etc.For yourself      - Boehringer-Ingelheim : anticoagulants        - Daiichi Sankyo : anticoagulants        - Bristol Myers Squibb : anticoagulants        - Medtronic : catheter ablation        - Johnson & Johnson : catheter ablation        - Boston Scientific : SICD Olshansky Brian 2020  Financial Declaration    Direct personal payment from healthcare industry: speaker fees, honoraria, consultancy, advisory board fees, investigator, committee member, etc.For yourself      - Lundbeck : Orthostatic hypotension Potpara Tatjana 2020  Financial Declaration    Payment from healthcare industry to your department or institution or any other body for your personal services: speaker fees, honoraria, consultancy, advisory board fees, investigator, committee member, etc.For yourself       - Bayer : Oral anticoagulation        - Pfizer : Oral anticoagulation Savelieva Irina 2020  Financial Declaration    Direct personal payment from healthcare industry: speaker fees, honoraria, consultancy, advisory board fees, investigator, committee member, etc.For yourself      - Menarini : Antianginal agent, potentially antiarrhythmic agent         - Gilead : Antianginal agent, potentially antiarrhythmic agent         - Pfizer : Oral anticoagulant        - Bayer AG : Oral anticoagulant        - Bristol Myers Squibb : Oral anticoagulant, potentially antiarrhythmic agent        - Richmond Pharmacology : Potentially antiarrhythmic agent (past) Stiles Martin 2020    Nothing to be declared Tamargo Juan Luis 2020    Nothing to be declared Ungar Andrea 2020    Nothing to be declared	
Anker Stefan	2020	Financial Declaration
		Direct personal payment from healthcare industry: speaker fees, honoraria, consultancy, advisory board fees, investigator, committee member, etc.For yourself      - BRAHMS GmbH : Biomarker in HF research
		- Abbott : COVID-19 SAB
		- Janssen / J&J : HF consultancy
		- Cordio : HF consultancy
		- Servier : HF registry steering committee
		- Boehringer-Ingelheim : HF trial executive committee (EMPEROR, EMPERIAL)
		- Vifor International : HF trial executive committees (AFFIRM-HF, DIAMOND-HF)
		- Cardiac Dimensions : HF trial steering committee
		- Bioventrix : HF trial steering committee
		- V-Wave : HF trial steering committee
		- Bayer AG : HF trial steering committee (DKD)
		- Astra Zeneca : SAB in HF (Germany)
		- Respicardia : Sleep apnea SAB.
		Research funding from healthcare industry under your direct/personal responsibility (to department or institution).For yourself      - Vifor International : CHF therapy development, FAIR-HFpEF & Fair-HF2
		- Abbott Vascular : CHF therapy development, RESHAPE-HF2
		Other Positions of Influence
		Any other interest (financial or otherwise) that should be declared in view of holding an ESC position.For yourself      - Fees for SAB-chair work (<10k EUR / yr) from Actimed Therapeutics, which is a research company (topic: cancer cachexia). I also hold founder's shares of Actimed Therapeutics (<20%).
Benetos Athanase	2020	Financial Declaration
		Direct personal payment from healthcare industry: speaker fees, honoraria, consultancy, advisory board fees, investigator, committee member, etc.For yourself      - Novartis : Dementia
Boriani Giuseppe	2020	Financial Declaration
		Direct personal payment from healthcare industry: speaker fees, honoraria, consultancy, advisory board fees, investigator, committee member, etc.For yourself      - Bayer : Anticoagulants
		- Boston Scientific : Pacemaker, cardioverter defibrillators
		- Medtronic : Pacemaker, cardioverter defibrillators
Bunch T Jared	2020	Financial Declaration
		Research funding from healthcare industry under your direct/personal responsibility (to department or institution).For yourself      - Boehringer-Ingelheim : Atrial Fibrillation
		- Boston Scientific : Atrial Fibrillation
		- Altathera : Atrial Fibrillation
		Other Positions of Influence
		Membership or affiliation in political, advocacy or patients organisations working in the field of cardiology.For yourself      - Heart Rhythm Society, American College of Cardiology, American Heart Association
Dagres Nikolaos	2020	
		Nothing to be declared
Dubner Sergio Jose	2020	Financial Declaration
		Direct personal payment from healthcare industry: speaker fees, honoraria, consultancy, advisory board fees, investigator, committee member, etc.For yourself      - Boehringer-Ingelheim : NOACs
		Payment from healthcare industry to your department or institution or any other body for your personal services: speaker fees, honoraria, consultancy, advisory board fees, investigator, committee member, etc.For yourself       - Johnson & Johnson : Fellow grant
Fauchier Laurent	2020	Financial Declaration
		Direct personal payment from healthcare industry: speaker fees, honoraria, consultancy, advisory board fees, investigator, committee member, etc.For yourself      - Bayer : Healthcare
		- Boehringer-Ingelheim : Healthcare
		- Medtronic : Healthcare
		- Novartis : Healthcare
		- BMS/Pfizer : Healthcare
		- XO : Healthcare
Ferrucci Luigi	2020	
		Nothing to be declared
Fumagalli Stefano	2020	Financial Declaration
		Direct personal payment from healthcare industry: speaker fees, honoraria, consultancy, advisory board fees, investigator, committee member, etc.For yourself      - Bruno Farmaceutici : Atrial Fibrillation
		- Bayer : Atrial Fibrillation and DOACs
		- Boehringer-Ingelheim : Atrial Fibrillation and DOACs
		- Pfizer : Atrial Fibrillation and DOACs
		- Bristol Myers Squibb : Atrial Fibrillation and DOACs
		- Daiichi Sankyo : Atrial Fibrillation, DOACs and Frailty
		Travel and meeting support from healthcare industry, independent of the above activities.For yourself      - Daiichi Sankyo : Geriatric Cardiology
Israel Carsten W	2020	Financial Declaration
		Direct personal payment from healthcare industry: speaker fees, honoraria, consultancy, advisory board fees, investigator, committee member, etc.For yourself      - Berlin Chemie AG : Antiarrhythmic drugs
		- BMS-Pfizer : Apixaban
		- Astra Zeneca : Brillique
		- Impulse Dynamics : Cardiac Contractility Management
		- Boehringer-Ingelheim : Dabigatran
		- Sanofi Aventis : Dronedarone
		- Daiichi Sankyo : Edoxaban
		- Servier : Ivabradin
		- Zoll Medical : Life Vest
		- Boston Scientific : Pacemakers, ICDs
		- Biotronik : Pacemakers, ICDs, CRT
		- Microport : Pacemakers, ICDs, CRT
		- Medtronic : Pacemakers, ICDs, CRT, ILR
		- Abbott : Pacemakers, ICDs, CRT, Occluder
		- Bayer Healthcare : Rivaroxaban
		Payment from healthcare industry to your department or institution or any other body for your personal services: speaker fees, honoraria, consultancy, advisory board fees, investigator, committee member, etc.For yourself       - Astra Zeneca : Brillique
		- Zoll Medical : Life Vest
		- Medtronic : Pacemakers, ICDs, CRT, ILR
		Travel and meeting support from healthcare industry, independent of the above activities.For yourself      - Sanofi Aventis : Antiarrhythmics
		- Berlin Chemie AG : Antiarrhythmics
		- Boehringer-Ingelheim : oral anticoagulation
		- Daiichi Sankyo : oral anticoagulation
		- Pfizer : oral anticoagulation
		- Bayer Healthcare : oral anticoagulation
		- Bristol Myers Squibb : oral anticoagulation
		- Abbott : Pacemaker, ICD, CRT
		- Boston Scientific : Pacemaker, ICD, CRT
		- Medtronic : Pacemaker, ICD, CRT
		- Biotronik : Pacemaker, ICD, CRT
		- Microport : Pacemaker, ICD, CRT
		- Zoll Medical : sudden cardiac death
		Research funding from healthcare industry under your direct/personal responsibility (to department or institution).For yourself      - Abbott Vascular : LAA occluders
		- Microport : Pacemaker, ICD, CRT
		Other Positions of Influence
		Membership or affiliation in political, advocacy or patients organisations working in the field of cardiology.For yourself      - Deutsche Herzstiftung
		Any other interest (financial or otherwise) that should be declared in view of holding an ESC position.For yourself      - Member of the Board of the World Society of Arrhythmias Editor of "Herzschrittmachertherapie und Elektrophysiologie"
Kamel Hooman	2020	Other Positions of Influence
		Any other interest (financial or otherwise) that should be declared in view of holding an ESC position.For yourself      - Dr. Kamel serves as a PI for the NIH-funded ARCADIA trial (NINDS U01NS095869) which receives in-kind study drug from the BMS-Pfizer Alliance for Eliquis® and ancillary study support from Roche Diagnostics, serves as Deputy Editor for JAMA Neurology, serves as a steering committee member of Medtronic’s Stroke AF trial (uncompensated), and serves on an endpoint adjudication committee for a trial of empagliflozin for Boehringer-Ingelheim.
Kenny Rose Anne	2020	Financial Declaration
		Direct personal payment from healthcare industry: speaker fees, honoraria, consultancy, advisory board fees, investigator, committee member, etc.For yourself      - Medtronic : Syncope
Lane Deirdre	2020	Financial Declaration
		Direct personal payment from healthcare industry: speaker fees, honoraria, consultancy, advisory board fees, investigator, committee member, etc.For yourself      - Boehringer-Ingelheim : Atrial fibrillation
		- Pfizer : Atrial fibrillation
		- Bayer Healthcare : Atrial fibrillation
		Research funding from healthcare industry under your direct/personal responsibility (to department or institution).For yourself      - Bristol Myers Squibb : Atrial fibrillation and stroke prevention
Lip Gregory Yh	2020	Financial Declaration
		Payment from healthcare industry to your department or institution or any other body for your personal services: speaker fees, honoraria, consultancy, advisory board fees, investigator, committee member, etc.For yourself       - Daiichi-Sankyo : Anticoagulation
		- Bayer/Janssen : Anticoagulation
		- Boehringer Ingelheim : Anticoagulation; Registries; Steering Committees
		- Pfizer : Anticoagulation; Registries
		- BMS : Antithrombotic therapy
		Research funding from healthcare industry under your direct/personal responsibility (to department or institution).For yourself      - Boehringer-Ingelheim : AF registries [unrestricted educational grant]
		Other Positions of Influence
		Any other interest (financial or otherwise) that should be declared in view of holding an ESC position.For yourself      - Shares in private limited company (a legal separate entity in UK), but no salary/dividends/income/personal renumeration received.
Marchionni Niccolo'	2020	Financial Declaration
		Direct personal payment from healthcare industry: speaker fees, honoraria, consultancy, advisory board fees, investigator, committee member, etc.For yourself      - Bayer : Direct Anticoagulants
		- Daiichi Sankyo : Direct Anticoagulants
		- Bristol Myers Squibb : Direct Anticoagulants
		Research funding from healthcare industry under your direct/personal responsibility (to department or institution).For yourself      - Bristol Myers Squibb : Direct Anticoagulants
Obel Isreal Wp	2020	
		Nothing to be declared
Okumura Ken	2020	Financial Declaration
		Direct personal payment from healthcare industry: speaker fees, honoraria, consultancy, advisory board fees, investigator, committee member, etc.For yourself      - Boehringer-Ingelheim : anticoagulants
		- Daiichi Sankyo : anticoagulants
		- Bristol Myers Squibb : anticoagulants
		- Medtronic : catheter ablation
		- Johnson & Johnson : catheter ablation
		- Boston Scientific : SICD
Olshansky Brian	2020	Financial Declaration
		Direct personal payment from healthcare industry: speaker fees, honoraria, consultancy, advisory board fees, investigator, committee member, etc.For yourself      - Lundbeck : Orthostatic hypotension
Potpara Tatjana	2020	Financial Declaration
		Payment from healthcare industry to your department or institution or any other body for your personal services: speaker fees, honoraria, consultancy, advisory board fees, investigator, committee member, etc.For yourself       - Bayer : Oral anticoagulation
		- Pfizer : Oral anticoagulation
Savelieva Irina	2020	Financial Declaration
		Direct personal payment from healthcare industry: speaker fees, honoraria, consultancy, advisory board fees, investigator, committee member, etc.For yourself      - Menarini : Antianginal agent, potentially antiarrhythmic agent
		- Gilead : Antianginal agent, potentially antiarrhythmic agent
		- Pfizer : Oral anticoagulant
		- Bayer AG : Oral anticoagulant
		- Bristol Myers Squibb : Oral anticoagulant, potentially antiarrhythmic agent
		- Richmond Pharmacology : Potentially antiarrhythmic agent (past)
Stiles Martin	2020	
		Nothing to be declared
Tamargo Juan Luis	2020	
		Nothing to be declared
Ungar Andrea	2020	
		Nothing to be declared
		
